# Synthesis of new representatives of A_3_B-type carboranylporphyrins based on *meso*-tetra(pentafluorophenyl)porphyrin transformations

**DOI:** 10.3762/bjoc.20.70

**Published:** 2024-04-12

**Authors:** Victoria M Alpatova, Evgeny G Rys, Elena G Kononova, Valentina A Ol'shevskaya

**Affiliations:** 1 A.N.Nesmeyanov Institute of Organoelement Compounds, Russian Academy of Sciences, 28, bld. 1 Vavilova street, 119334 Moscow, Russian Federation

**Keywords:** bioconjugation, carboranes, fluorine, porphyrin, S_N_Ar aromatic substitution

## Abstract

A carboranylporphyrin of A_3_B-type bearing a single pentafluorophenyl ring was prepared through the regioselective nucleophilic aromatic substitution reaction of the *p*-fluorine atoms in 5,10,15,20-tetrakis(pentafluorophenyl)porphyrin with 9-mercapto-*m*-carborane. The reaction of this porphyrin with sodium azide led to the selective substitution of the *p*-fluorine atom in the pentafluorophenyl substituent with an azide functionality which upon reduction with SnCl_2_ resulted in the formation of the corresponding porphyrin with an amino group. Pentafluorophenyl-substituted A_3_B-porphyrins were studied and transformed to thiol and amino-substituted compounds allowing for the preparation of porphyrins with different reactive groups such as hydroxy and amino derivatives capable for further functionalization and conjugation of these porphyrins to other substrates. In addition, conjugates containing maleimide or biotin entities in the structure of carborane A_3_B-porphyrin were also synthesized based on the amino-substituted A_3_B-porphyrin. The structures of the prepared carboranylporphyrins were determined by UV–vis, IR, ^1^H, ^19^F, ^11^B NMR spectroscopic data and MALDI mass spectrometry.

## Introduction

Porphyrins are available macroheterocyclic compounds which play an important role in diverse areas of scientific research owing to their unique photophysical, electrochemical, and optical properties [1]. They have been widely studied in biomedical applications, as biosensors, bioimaging probes, and especially as photosensitizers (PSs) in photodynamic therapy (PDT) [2]. PDT is a treatment modality that uses the combination of a non-toxic PS, oxygen, and light to treat diseases ranging from cancer to age-related macular degeneration and antibiotic-resistant infections [3–6]. Currently, there are a few photosensitizers approved for clinical PDT such as Photofrin^®^, Foscan^®^, Lutex^®^, Tookad^®^, Purlytin^®^, Visudyne^®^ and Laserphyrin^®^ [7] and experience in clinical use of PDT shows that this method belongs to one of promising directions in modern clinical oncology [8].

Further improvement of the PDT method requires the search for new photosensitizers having higher photoactivity, tumor selectivity, and high singlet oxygen quantum yield, as well as low in vivo toxicity [7]. Therefore, some strategies have been developed to enhance the therapeutic efficiency of tetrapyrrole compounds [9] since the delivery of a drug at a specific area in the body has vital importance to treat diseases. An alternative approach to solve this problem focused on the postfunctionalization of the porphyrin macrocycle with different linker groups capable for targeting conjugation of these porphyrins to other biological substrates and thus facilitate the conjugation with biomacromolecules [10–11]. The modification of the porphyrin periphery with amino-, azido-, epoxy-, hydroxy-, and maleimido-functionalities is usually used for the covalent linkage of the porphyrin to the targeted biomacromolecule [10–11]. In this context, fluorinated porphyrins have attracted considerable interest due to their biological properties such as low toxicity, metabolic stability, and cellular uptake. The introduction of a fluorine atom into the molecule is the feasibility to change drastically its biological properties and to modify the profile of biological activity due to optimum fluorine lipophylic properties, and enhanced interaction with lipid membranes [12–14]. Pentafluorophenyl-substituted porphyrin systems are especially useful for the connection of various functionalities capable for coupling with biomolecules via the nucleophilic aromatic (S_N_Ar) substitution reactions [15–16]. A variety of nucleophiles such as amines [17–18], alcohols [18–20], thiols [17,19,21–23], and carboranes [17,24–27] have been studied in selective S_N_Ar substitution reactions of the *p*-fluorine atoms in *meso*-pentafluorophenyl-substituted porphyrins. Carboranes, due to their unique physical and chemical properties such as high chemical and biological stability [28–29], three-dimensional aromaticity [30–31], low toxicity [28], high hydrophobicity, and enriched boron content [32–33] are perspective compounds in drug development [34–37]. Owing to their stability, carboranes also may increase the in vivo stability and bioavailability of pharmaceuticals that might otherwise rapidly metabolize [38]. The functionalization of porphyrins with carborane clusters provides dual-action photo(radio)sensitizers that are efficient for both PDT and boron neutron capture therapy (BNCT) [27,39]. The preparation of compounds with dual therapeutic efficiency is of great importance since they improve the therapeutic effect of sensitizer by the action on the different cellular sites. Here, we report the synthesis and characterization of tris(carboranyl)porphyrins of A_3_B-type (where “B” corresponds to the substituent responsible for bioconjugate coupling) based on the transformations of 5,10,15,20-tetrakis(pentafluorophenyl)porphyrin which was used as a basic compound for the synthesis of new boronated conjugates with functionalized linker groups suitable for bioconjugation or which may be efficient for PDT and BNCT improvement.

## Results and Discussion

### Synthesis

**Scheme 1:**
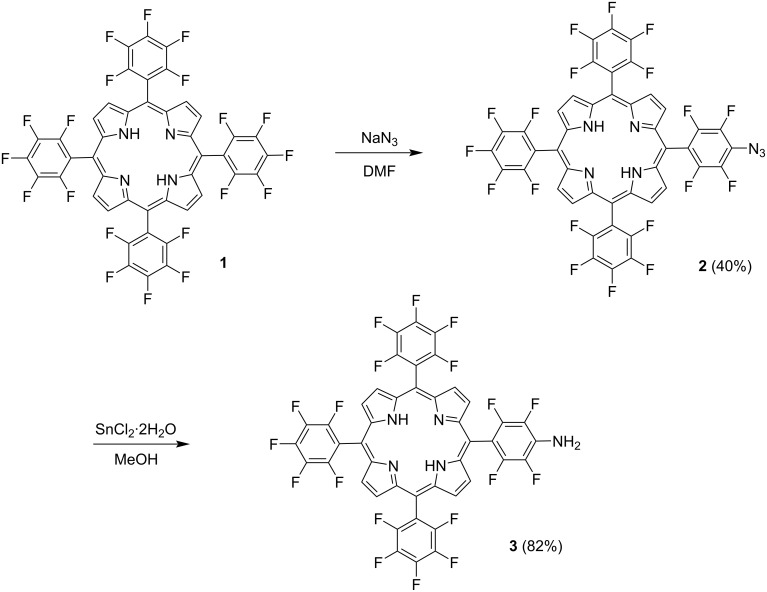
Synthesis of porphyrins **2** and **3**.

Nucleophilic substitution reactions of the four *p*-fluorine atoms in 5,10,15,20-tetrakis(pentafluorophenyl)porphyrin (**1**) are well studied [15–27]. In order to prepare boronated PSs of A_3_B-type the employed synthetic strategy included the preparation of monoazido-substituted tris(pentafluorophenyl)porphyrin **2** by the reaction of porphyrin **1** with sodium azide (molar ratio 1:1.9) in DMF at ambient temperature for 4 h. Under these reaction conditions, monoazide derivative **2** was obtained in 40% yield along with a mixture of porphyrin **1**, di- and triazido-substituted derivatives. The reaction mixture was separated by column chromatography on SiO_2_ using CH_2_Cl_2_/hexane 2:8 as an eluent. The reduction of the azide substituent in porphyrin **2** with SnCl_2_·2H_2_O in MeOH resulted in the formation of porphyrin amino-derivative **3** in 82% yield (Scheme 1). The molecular structures of compounds **2** and **3** were confirmed by a combination of NMR spectroscopy and mass spectrometry.

**Scheme 2:**
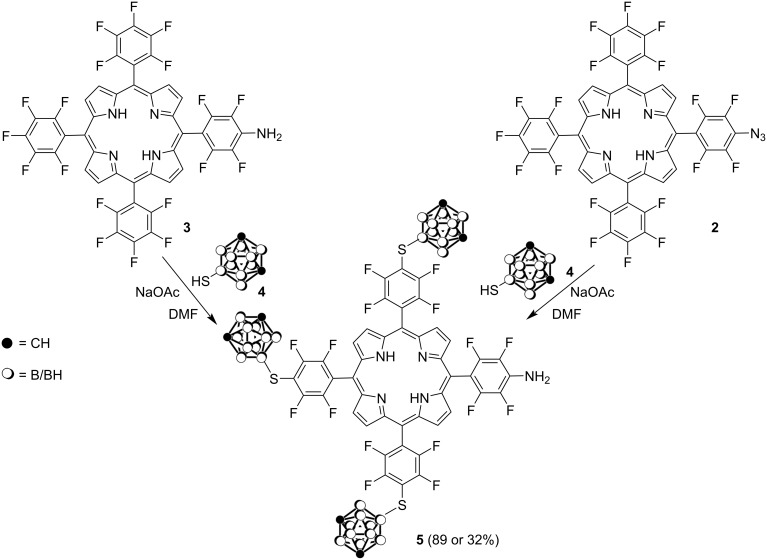
Synthesis of carborane aminoporphyrin **5**.

Having synthesized porphyrins **2** and **3** we next studied the modification of the pentafluorophenyl substituents with carborane clusters via the S_N_Ar substitution reaction with carborane nucleophiles [17,24–27]. These reactions are well studied for porphyrin **1** [17,24–27] to afford the corresponding carborane derivatives efficient in PDT and BNCT applications. The reaction of porphyrin **3** with 9-mercapto-*m*-carborane (**4**) readily proceeded in DMF in the presence of anhydrous NaOAc under argon atmosphere to give porphyrin derivative **5** in 89% yield (Scheme 2) containing three carborane polyhedra bound to the fluorophenylporphyrin substituents via the boron atom. At the same time the S_N_Ar substitution reaction for the azido-substituted porphyrin **2** with mercaptocarborane **4** also afforded the amino-substituted porphyrin **5** in 32% yield (Scheme 2). During the reaction the reduction of the azide group under the action of carboranethiol was observed which is consistent with literature data [40–41].

**Scheme 3:**
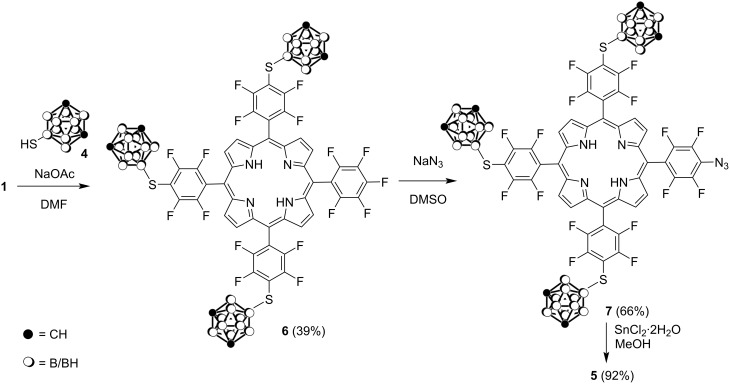
Synthesis of carboranyl-substituted porphyrins **5**–**7**.

To optimize the reaction conditions for the preparation of boronated porphyrin **5** we then performed the reaction of porphyrin **1** with mercaptocarborane **4** (molar ratio 1:4) in DMSO in the presence of anhydrous NaOAc for 1 h at ambient temperature under argon. Under these reaction conditions, the tris(carboranyl)-substituted porphyrin **6** was obtained in 39% yield after purification by column chromatography on SiO_2_ using CHCl_3_/hexane 1:1 as eluent (Scheme 3).

It should be noted that the reaction of porphyrin **6** with NaN_3_ in DMSO at 20 °C for 48 h resulted in a mixture of azidoporphyrin **7** and amino derivative **5** which were separated by column chromatography on SiO_2_ to give porphyrin **7** and porphyrin **5** in 66% and 33% yields, respectively. The reduction of porphyrin **7** with SnCl_2_·2H_2_O in MeOH afforded porphyrin **5** in 92% yield.

**Scheme 4:**
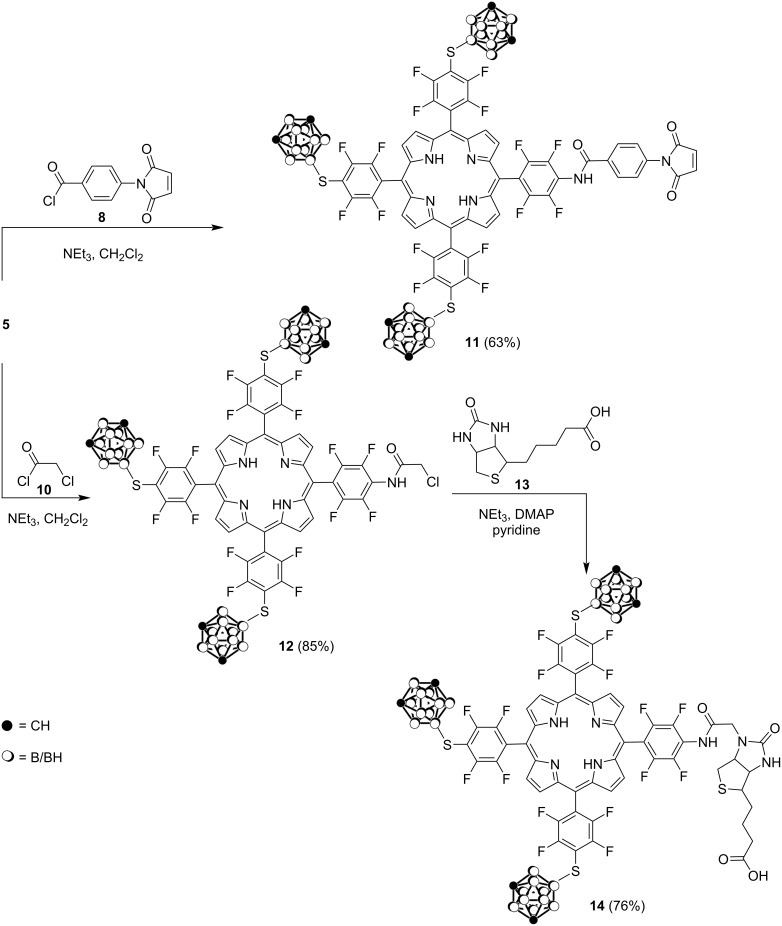
Synthesis of acylated carboranylporphyrins **11**, **12**, and **14**.

We investigated the ability of the amino group in porphyrin **5** to enter acylation reactions with 4-(*N*-maleimido)benzoyl chloride (**8**, prepared in situ from 4-(*N*-maleimido)benzoic acid (**9**) and oxalyl chloride) and chloroacetyl chloride (**10**) with the aim of using these compounds for further functionalization. The reactions were carried out in CH_2_Cl_2_ in the presence of Et_3_N (Scheme 4) to afford the acylated derivatives **11** and **12** in 63 and 85% yield, respectively. It is known [42–43] that maleimido-substituted compounds readily enter reactions with thiols to generate thiosuccinimide products and meanwhile this method has become one of the most popular route for the site-selective modification of cysteine residues in bioconjugation technology. We suppose that the maleimide group in porphyrin **11** is a useful target for thiol conjugation via Michael addition reactions [44]. This also concerns biotin-conjugated organic molecules which have been also used for selective delivery of the drug to cancer cells [45]. Here, biotin was conjugated to porphyrin **12** which was obtained by alkylation of the amino group in compound **5** with chloroacetyl chloride (**10**) to give porphyrin biotin conjugate **14** in 76% yield (Scheme 4).

**Scheme 5:**
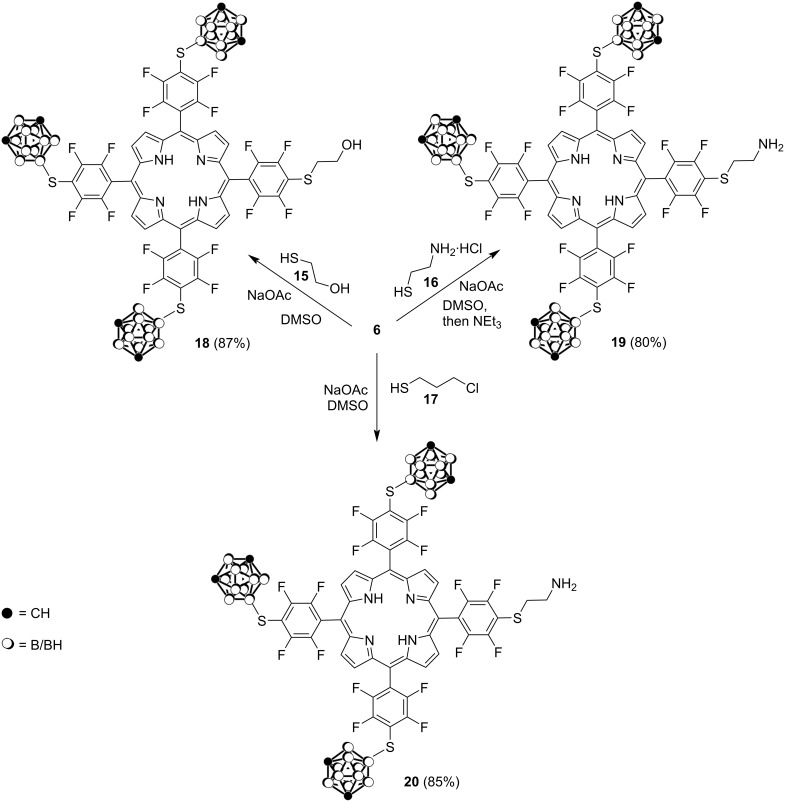
Synthesis of thio-substituted carboranylporphyrins **18**–**20**.

We also studied the nucleophilic substitution reactions of the *p*-fluorine atom in the pentafluorophenyl-containing porphyrin **6** with thiol-substituted compounds such as 2-mercaptoethanol (**15**), cysteamine hydrochloride (**16**), and 3-chloro-1-propanethiol (**17**) as shown in Scheme 5.

The reactions proceeded readily in DMSO at room temperature for 10 min using anhydrous NaOAc as a base to afford the corresponding boronated porphyrin conjugates **18**–**20** in 80–87% yields.

**Scheme 6:**
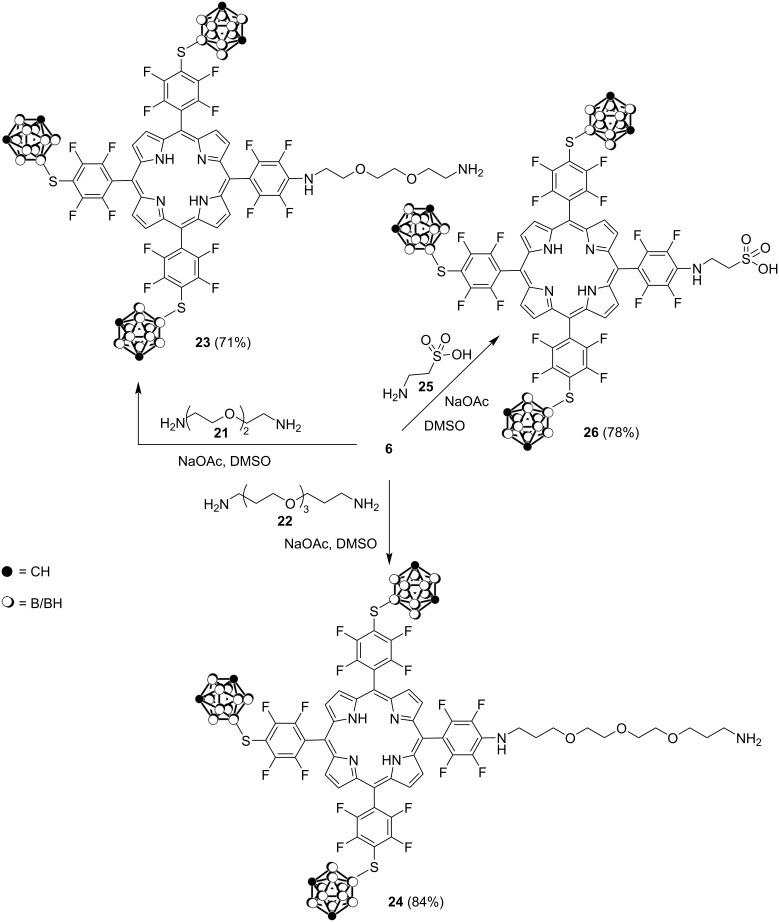
Synthesis of amino-substituted carboranylporphyrins **23**, **24**, and **26**.

Exploring the reactivity of the *p*-fluorine atom similar nucleophilic substitution reactions of porphyrin **6** were carried out with 1,8-diamino-3,6-dioxaoctane (**21**) and 1,13-diamino-4,7,10-trioxatridecane (**22**) in DMSO at 70 °C for 30 min to form amino-conjugates **23** and **24** in 71 and 84% yield, respectively, containing ethylene glycol linkers with terminal primary amino groups (Scheme 6). The presence of ethylene glycol residues in bioactive molecules is known to enhance the aqueous solubility and tumor selectivity of hydrophobic drugs through the enhanced permeability and retention effect [46]. It was also shown that porphyrin **6** undergoes reaction with taurine (2-aminoethanesulfonic acid, **25**) which is an essential nutraceutical with diverse cytoprotective and therapeutic actions. It is synthesized from cysteine and is excreted without any further metabolism [47]. The reaction of taurine (**25**) with porphyrin **6** proceeded in DMSO at 20 °C for 72 h to afford taurine-containing conjugate **26** in 78% yield (Scheme 6).

Conjugates **19**, **23**, **24**, and **26** can be easily converted into hydrophilic charged entities by the protonation of the unsubstituted amino functionalities in their structure providing improved bioconjugation.

### Spectroscopic data

All porphyrin conjugates were structurally characterized by IR, UV–vis, NMR spectroscopy, and mass spectrometry. The IR spectra of porphyrins **2** and **3** exhibit the absorption band at 3321 cm^−1^ corresponded to NН stretching vibrations. Bands at 2127 cm^−1^ confirmed the presence of the N_3_ group in porphyrins **2** and **7**. The IR spectra of porphyrins **5**–**7**, **11**, **12**, **14**, **18**–**20**, **23**, **24**, and **26** exhibit absorption bands at 2605–2609 cm^−1^ assigned to the BH-stretching vibration in neutral *closo*-carborane polyhedra and the bands at 3061–3069 cm^−1^ related to carborane CH groups. All prepared porphyrins **2**, **3**, **5**–**7**, **11**, **12**, **14**, **18**–**20**, **23**, **24**, and **26** had the characteristic bands at ν = 1466–1499 cm^−1^ assigned to C–F stretching vibrations. Bands in the 1797–1641 cm^−1^ range in porphyrins **11**, **12**, and **14** correspond to the displacement of the C=O group. In the ^1^H NMR spectra eight β-protons of the porphyrin macrocycle for all compounds **2**, **3**, **5**–**7**, **11**, **12**, **14**, **18**–**20**, **23**, **24**, and **26** were found between δ = 8.94–9.39 ppm and broadened singlets of the porphyrin inner NH protons were observed at δ = −2.83 to −3.16 ppm. The signals of the carborane CH protons in porphyrins **5**–**7**, **11**, **12**, **14**, **18**–**20**, **23**, **24**, and **26** were observed at δ = 3.83–4.14 ppm. The expected signals with appropriate multiplicities for the functionalities linked at the pentafluorophenyl substituent of porphyrins **3**, **5**, **11**, **12**, **14**, **18**–**20**, **23**, **24**, and **26** were also observed supporting the structures of these compounds (see Supporting Information File 1, experimental and Figures S1–S14 for details). The ^19^F NMR spectra were also in good agreement with the structures of the synthesized compounds and the data are given in Table 1.

**Table 1:** Chemical shifts (ppm) and multiplicities (*J*, Hz) in ^19^F NMR spectra for all synthesized compounds.

compound	*o*-fluorine	*p*-fluorine	*m*-fluorine

**2**	−136.5 (d, 19.2, 6F), −137.1 (dd, 22.0, 8.3, 2F)	−151.2 (t, 19.2 , 3F)	−151.5 (dd, 22.0, 11.0, 2F), −161.3 (t, 19.2, 6F)
**3**	−136.5 (d, 19.2, 6F), −140.5 (d, 16.5, 2F)	−151.5 (dd, 38.5, 19.2, 3F)	−161.5 (t, 16.3, 6F), −161.9 (d, 13.7, 2F)
**5**	−133.8 (dd, 24.7, 13.7, 6F), −144.1 (d, 16.5, 2F),	–	−139.7 (dd, 24.7, 13.7, 6F), −164.0 (d, 16.5 Hz, 2F)
**6**	−133.7 (dd, 24.7, 13.7, 6F), −139.8 (dd, 22.0, 5.5, 2F)	−155.4 (t, 22.0, 1F)	−139.6 (dd, 27.5, 13.7, 6F), −164.4 (td, 22.0, 13.7, 2F)
**7**	−133.7 (dd, 25.2, 13.8, 6F), −140.7 (dd, 21.8, 12.6, 2F)	–	−139.6 (dd, 25.2, 13.8, 6F), −153.84 (dd, 21.8, 12.6, 2F)
**11**	−133.7 (dd, 25.2, 14.9, 6F), −141.0 (dd, 23.0, 13.6, 2F)	–	−139.5 (dd, 25.2, 14.9, 6F), −146.3 (dd, 23.0, 13.7, 2F)
**12**	−133.6 (dd, 25.2, 13.8, 6F), −140.8 (dd, 22.9, 12.6, 2F)	–	−139.5 (dd, 25.2, 13.8, 6F), −146.4 (dd, 22.9, 12.6, 2F)
**14**	−132.9 (dd, 26.8, 11.5, 6F), −140.3 (d, 18.7 Hz, 2F)	–	−138.7 (dd, 26.8, 11.5, 6F), −144.2 (d, 16.5 Hz, 2F)
**18**	−133.8 (dd, 24.7, 13.7, 6F), −135.7 (dd, 24.7, 13.7, 2F)	–	−139.7 (dd, 27.5, 13.7, 6F), −140.2 (dd, 24.7, 13.7, 2F)
**19**	−133.7 (dd, 24.1, 13.8, 6F), −135.6 (dd, 25.2, 14.9, 2F)	–	−139.6 (dd, 25.2, 12.6, 6F), −140.3 (dd, 26.4, 13.8, 2F)
**20**	−133.7 (dd, 24.8, 13.8, 6F), −135.6 (dd, 24.8, 13.8, 2F)	–	−139.7 (dd, 24.8, 13.8, 8F)
**23**	−133.8 (dd, 18.4, 6.9, 6F), −143.5 (dd, 22.2, 9.2, 2F)	–	−139.7 (dd, 26.4, 13.8, 6F), −161.7 (dd, 25.2, 4.6, 2F)
**24**	−133.8 (dd, 25.2, 13.8, 6F), −143.7 (dd, 19.7, 11.5, 2F)	–	−139.7 (dd, 25.2, 13.8, 6F), −161.7 (dd, 19.7, 11.5, 2F)
**26**	−129.8 (dd, 25.2, 13.8, 6F), −139.2 (d, 17.2, 2F)	–	−135.6 (dd, 25.2, 14.98, 6F), −158.3 (d, 14.9, 2F)

The ^11^B NMR signals of compounds **5**–**7**, **11**, **12**, **14**, **18**–**20**, **23**, **24**, and **26** are in the range from δ = −0.9 to −17.0 ppm confirming the *closo*-structure of the carborane polyhedra.

## Conclusion

In this article a synthesis of A_3_B-type carboranylporphyrins as potential photosensitizers for PDT was developed based on the detailed study of the functionalization of a single pentafluorophenyl substituent in 5,10,15,20-tetrakis(pentafluorophenyl)porphyrin with azido or amino functional groups. These compounds were used as a platform for the design of A_3_B-type carboranylporphyrins by the S_N_Ar substitution reactions with 9-mercapto-*m*-carborane. As a result, tris(carboranyl)-substituted porphyrins containing pentafluorophenyl- or *p*-aminotetrafluorophenyl-substituents were synthesized and used in the reactions with a variety of thio- or amino-nucleophiles to form functionalized linkers capable to connect these porphyrins with biomolecules, thus improving their biomedical characteristics and theraputic efficacy for PDT and BNCT due to the combination of different substituents within porphyrin framework. Amide coupling of A_3_B-type carboranylporphyrin containing an amino functionality was supported by the design of conjugates containing maleimide and biotin substituents. The structures of prepared carboranylporphyrins were determined by UV–vis, IR, ^1^H ^19^F, ^11^B NMR spectroscopic data and MALDI mass spectrometry.

## Supporting Information

File 1 Experimental details and characterization data.

## Data Availability

All data that supports the findings of this study is available in the published article and/or the supporting information to this article.
