# Supplementary material for: Synthesis of new representatives of A3B-type carboranylporphyrins based on meso-tetra(pentafluorophenyl)porphyrin transformations
Source: Beilstein J Org Chem. 2024 Apr 12;20:767–76. doi: 10.3762/bjoc.20.70 (PMC11022374; doi:10.3762/bjoc.20.70)
Supplement: File 1 — Experimental details and characterization data. [file Beilstein_J_Org_Chem-20-767-s001.pdf]

BEILSTEIN JOURNAL OF ORGANIC CHEMISTRY

Supporting Information

for

Synthesis of new representatives of A_3B-type carboranylporphyrins based on *meso*-tetra(pentafluorophenyl)porphyrin transformations

Victoria M. Alpatova, Evgeny G. Rys, Elena G. Kononova and Valentina A. Ol'shevskaya

Beilstein J. Org. Chem. **2024,** *20,* 767–776. doi:10.3762/bjoc.20.70

Experimental details and characterization data

License and Terms: This is a supporting information file under the terms of the Creative Commons Attribution License (https://creativecommons.org/licenses/by/4.0). Please note that the reuse, redistribution and reproduction in particular requires that the author(s) and source are credited and that individual graphics may be subject to special legal provisions.
The license is subject to the *Beilstein Journal of Organic Chemistry* terms and conditions: (https://www.beilstein-journals.org/bjoc/terms)

Table of contents

1. Synthesis (general remarks) S4
2. Synthesis and characterization of new A_3B-type porphyrins S4
3. The ^{1}H and ^{19}F NMR spectra of 5-(4-azido-2,3,5,6-tetrafluorophenyl)-10,15,20-tris(pentafluorophenyl)porphyrin (**2**) S13
4. The ^{1}H and ^{19}F NMR spectra of 5-(4-amino-2,3,5,6-tetrafluorophenyl)-10,15,20-tris(pentafluorophenyl)porphyrin (**3**) S14
5. The ^{1}H, ^{19}F and ^{11}B NMR spectra of 5-[4-amino-2,3,5,6-tetrafluorophenyl]-10,15,20-tris{[4-(*m*-carboran-9'-yl)thio]-2,3,5,6-tetrafluorophenyl}porphyrin (**5**) S15
6. The ^{1}H, ^{19}F and ^{11}B NMR spectra of 5, 10, 15-tris{[4-(*m*-carboran-9'-yl)thio]-2,3,5,6-tetrafluorophenyl}-20-(2,3,4,5,6-pentafluorophenyl]porphyrin (**6**) S16
7. The ^{1}H, ^{19}F and ^{11}B NMR spectra of 5-[4-azido-2,3,5,6-tetrafluorophenyl]-10,15,20-tris{[4-(*m*-carboran-9'-yl)thio]-2,3,5,6-tetrafluorophenyl}porphyrin (**7**) S17
8. The ^{1}H, ^{19}F and ^{11}B NMR spectra of 5,10,15-tris{[(*m*-carboran-9'-yl)thio]-2,3,5,6-tetrafluorophenyl}-20-{[4-(2',5'-dioxo-2',5'-dihydro-1*H*-pyrrol-1'-yl)benzamido]-2,3,5,6-tetrafluorophenyl}porphyrin (**11**) S18
9. The ^{1}H, ^{19}F and ^{11}B NMR spectra of 5,10,15-tris{[4,4,4-(*m*-carboran-9'-yl)thio]-2,3,5,6-tetrafluorophenyl}-20-[4-(2'-chloroacetamido)-2,3,5,6-tetrafluorophenyl]porphyrin (**12**) S19
10. The ^{1}H, ^{19}F and ^{11}B NMR spectra of 5,10,15-tris{[4,4,4-(*m*-carboran-9'-yl)thio]-2,3,5,6-tetrafluorophenyl}-20-[4-(2'-(4'-(4'-carboxybutyl)-2-oxohexahydro-*1H*-thieno[3,4-d]imidazol-1'-yl)acetamido)-2,3,5,6-tetrafluorophenyl]porphyrin (**14**) S20
11. The ^{1}H, ^{19}F and ^{11}B spectra of 5,10,15-tris{[(*m*-carboran-9'-yl)thio]-2,3,5,6-tetrafluorophenyl}-20-{[(4-(2'-hydroxyethyl)thio]-2,3,5,6-tetrafluorophenyl}porphyrin (**18**) S21
12. The ^{1}H, ^{19}F and ^{11}B spectra of 5-{[4-(2'-aminoethyl)thio]-2,3,5,6-tetrafluorophenyl}-10,15,20-tris{[4,4,4-(*m*-carboran-9'-yl)thio]-2,3,5,6- S22

tetrafluorophenyl}porphyrin (**19**)

13. The ^{1}H, ^{19}F and ^{11}B spectra of 5,10,15-tris{[4,4,4-(*m*-carboran-9’-yl)thio]-2,3,5,6-tetrafluorophenyl}-20-{[(4-(3’-chloropropyl)thio]-2,3,5,6-tetrafluorophenyl]porphyrin (**20**) S23
14. The ^{1}H, ^{19}F and ^{11}B spectra of 5-[(4-(2’-(2’-(2’-aminoethoxy)ethoxy)ethyl)amino)-2,3,5,6-tetrafluorophenyl]-10,15,20-tris{[4,4,4-(*m*-carboran-9’-yl)thio]-2,3,5,6-tetrafluorophenyl}porphyrin (**23**) S24
15. The ^{1}H, ^{19}F and ^{11}B spectra of 5-[(4-(((3’-(2’-(2’-(3’-aminopropoxy)ethoxy)ethoxy)propyl)amino)-2,3,5,6-tetrafluorophenyl]-10,15,20-tris{[4,4,4-(*m*-carboran-9’-yl)thio]-2,3,5,6-tetrafluorophenyl}porphyrin (**24**) S25
16. The ^{1}H, ^{19}F and ^{11}B spectra of 5,10,15-tris{[4,4,4-(*m*-carboran-9’-yl)thio]-2,3,5,6-tetrafluorophenyl}-20-[4-((2’-sulfoethyl)amino)-2,3,5,6-tetrafluorophenyl]porphyrin (**26**) S26
17. MALDI-TOF mass spectrum for 5-(4-azido-2,3,5,6-tetrafluorophenyl)-10,15,20-tris(pentafluorophenyl)porphyrin (**2**) S27
18. MALDI-TOF mass spectrum for 5-(4-amino-2,3,5,6-tetrafluorophenyl)-10,15,20-tris(pentafluorophenyl)porphyrin (**3**) S28
19. MALDI-TOF mass spectrum for 5-[4-amino-2,3,5,6-tetrafluorophenyl]-10,15,20-tris{[4-(*m*-carboran-9’-yl)thio]-2,3,5,6-tetrafluorophenyl}porphyrin (**5**) S28
20. MALDI-TOF mass spectrum for 5, 10, 15-tris{[4-(*m*-carboran-9’-yl)thio]-2,3,5,6-tetrafluorophenyl}-20-(2,3,4,5,6-pentafluorophenyl]porphyrin (**6**) S29
21. MALDI-TOF mass spectrum for 5-[4-azido-2,3,5,6-tetrafluorophenyl]-10,15,20-tris{[4-(*m*-carboran-9’-yl)thio]-2,3,5,6-tetrafluorophenyl}porphyrin (**7**) S29
22. MALDI-TOF mass spectrum for 5,10,15-tris{[(*m*-carboran-9’-yl)thio]-2,3,5,6-tetrafluorophenyl}-20-{[4-(2’,5’-dioxo-2’,5’-dihydro-1*H*-pyrrol-1’-yl)benzamido]-2,3,5,6-tetrafluorophenyl}porphyrin (**11**) S30
23. MALDI-TOF mass spectrum for 5,10,15-tris{[4,4,4-(*m*-carboran-9’-yl)thio]-2,3,5,6-tetrafluorophenyl}-20-[4-(2’-chloroacetamido)-2,3,5,6- S30

tetrafluorophenyl]porphyrin (**12**)

24. MALDI-TOF mass spectrum for 5,10,15-tris{[4,4,4-(*m*-carboran-9’-yl)thio]-2,3,5,6-tetrafluorophenyl}-20-[4-(2’-(4’-(4’-carboxybutyl)-2-oxohexahydro-*1H*-thieno[3,4-d]imidazol-1’-yl)acetamido)-2,3,5,6-tetrafluorophenyl]porphyrin (**14**) S31
25. MALDI-TOF mass spectrum for 5,10,15-tris{[(*m*-carboran-9’-yl)thio]-2,3,5,6-tetrafluorophenyl}-20-{[(4-(2’-hydroxyethyl)thio]-2,3,5,6-tetrafluorophenyl}porphyrin (**18**) S31
26. MALDI-TOF mass spectrum for 5-{[4-(2’-aminoethyl)thio]-2,3,5,6-tetrafluorophenyl}-10,15,20-tris{[4,4,4-(*m*-carboran-9’-yl)thio]-2,3,5,6-tetrafluorophenyl}porphyrin (**19**) S32
27. MALDI-TOF mass spectrum for 5,10,15-tris{[4,4,4-(*m*-carboran-9’-yl)thio]-2,3,5,6-tetrafluorophenyl}-20-{[(4-(3’-chloropropyl)thio]-2,3,5,6-tetrafluorophenyl]porphyrin (**20**) S32
28. MALDI-TOF mass spectrum for 5-[(4-(2’-(2’-(2’-aminoethoxy)ethoxy)ethyl)amino)-2,3,5,6-tetrafluorophenyl]-10,15,20-tris{[4,4,4-(*m*-carboran-9’-yl)thio]-2,3,5,6-tetrafluorophenyl}porphyrin (**23**) S33
29. MALDI-TOF mass spectrum for 5-[(4-(((3’-(2’-(2’-(3’-aminopropoxy)ethoxy)ethoxy)propyl)amino)-2,3,5,6-tetrafluorophenyl]-10,15,20-tris{[4,4,4-(*m*-carboran-9’-yl)thio]-2,3,5,6-tetrafluorophenyl}porphyrin (**14**) S33
30. MALDI-TOF mass spectrum for 5,10,15-tris{[4,4,4-(*m*-carboran-9’-yl)thio]-2,3,5,6-tetrafluorophenyl}-20-[4-((2’-sulfoethyl)amino)-2,3,5,6-tetrafluorophenyl]porphyrin (**26**) S33
31. References S33

1. Synthesis (general remarks)

General remarks on instrumentation and chemicals

All reactions were performed in an atmosphere of dry argon. All chemicals were commercially available and used without further purification. 5,10,15,20-Pentafluorophenylporphyrin (**1**) was synthesized according to the reported procedure [1]. Mercaptocarborane **4** was prepared according to the published data [2]. The solvents were purified by conventional methods before being used. DMSO HPLC was used as a solvent. ^{1}H, ^{11}B, and ^{19}F NMR spectra were recorded on a Bruker Avance-400 spectrometer operating at 400.13 MHz for ^{1}H NMR, 128.38 MHz for ^{11}B NMR, and 376.50 MHz for ^{19}F NMR. ^{1}H and ^{19}F NMR spectra for compounds **7**, **11**, **12**, **14**, **19**, **23**, **24**, and **26** were recorded on a Bruker Avance-300 spectrometer operating at 300.13 MHz for ^{1}H NMR and 282.40 MHz for ^{19}F NMR. Chemical shifts (δ) were referenced to the residual solvent peak ($CDCl_3$, ^{1}H: 7.26 ppm; acetone-d_6, ^{1}H: 2.05 ppm; THF-d_8, ^{1}H: 1.73, 3.58 ppm; $(CD_3)_2SO$ ^{1}H: 2.50 ppm) for ^{1}H, external $BF_3{\cdot}OEt_2$ for ^{11}B and external $CFCl_3$ for ^{19}F. IR spectra were recorded on a Bruker FTIR spectrometer Tensor 37 in KBr pellets. The UV–vis spectra were measured on a computerized spectrophotometer Specord M 40 Carl Zeiss in CH_2Cl_2, $(CH_3)_2CO$ or THF. The course of reactions and purity of compounds were monitored by TLC using Sorbfil plates. Merck silica gel L 0.040–0.060 mesh was used for column chromatography. The MALDI TOF mass spectra were recorded with a Shimadzu Axima Confidence time-of-flight spectrometer (Shimadzu Biotech, Kyoto, Japan) in the reflectron high resolution mode with a nitrogen laser (λ = 337 nm). Positive ions were registered. The mass range between 200 and 2000 was scanned. DCTB was used as a matrix.

2. Synthesis and characterization of new A_3B-type porphyrins

5-(4-Azido-2,3,5,6-tetrafluorophenyl)-10,15,20-tris(pentafluorophenyl)porphyrin (2)

A mixture of porphyrin **1** (400 mg, 0.41 mmol) and sodium azide (50 mg, 0.77 mmol) in DMF (8 mL) was stirred at room temperature under argon for 4 h. The obtained mixture was poured into water (100 mL), extracted with CH_2Cl_2 (3 × 30 mL), dried over Na_2SO_4, and filtered. After evaporation of the solvent, the residue was purified by column chromatography on silica gel, using hexane–CH_2Cl_2 8:2 as an eluent to afford azidoporphyrin **2** (165 mg, 40%). IR (KBr) ν_{max}, cm^{-1}: 3321 (NH), 2127 (N_3), 1498 (CF). UV-vis (CH_2Cl_2): λ_{max} [nm] (logε) = 412 (5.02), 506 (3.81), 584 (3.40). ^{1}H NMR (400.13 MHz,

$CDCl_3$), δ: –2.90 (br s, 2H, porphyrin NH), 8.94 (br s, 6H, β-H), 8.96 (br s, 2H, β-H). ^{19}F NMR (376.5 MHz, $CDCl_3$), δ: –136.5 (d, *J* = 19.2 Hz, 6F), –137.1 (dd, *J* = 22.0, 8.3Hz, 2F), –151.2 (t, *J* = 19.2 Hz, 3F), –151.5 (dd, *J* = 22.0, 11.0 Hz, 2F), –161.3 (t, *J* = 19.2 Hz, 6F). MALDI-MS: *m/z* found 997.84 $[M]^+$, calcd. for $C_{44}H_{10}F_{19}N_7$ 997.57.

5-(4-Amino-2,3,5,6-tetrafluorophenyl)-10,15,20-tris(pentafluorophenyl)porphyrin (3)

A mixture of porphyrin **2** (1010 mg, 1.01 mmol) and $SnCl_2{\cdot}2H_2O$ (685 mg, 3.04 mmol) in dry MeOH (30 mL) was stirred at room temperature under argon for 10 h. After completion of the reaction (TLC control), MeOH was evaporated and the residue was purified by column chromatography on silica gel, using hexane–CH_2Cl_2 6:4 as an eluent to give 983 mg (82%) of aminoporphyrin **3**. UV-vis (CH_2Cl_2): λ_{max} [nm] (log*ε*) = 413 (5.16), 507 (3.98), 584 (3.53). IR (KBr) ν_{max}, cm^{-1}: 3321 (NH), 1499 (CF). ^1H NMR (400.13 MHz, $CDCl_3$), δ: –2.86 (br s, 2H, porphyrin NH), 4.50 (br s, 2H, NH_2), 8.95 (br s, 6H, β-H), 9.06 (br s, 2H, β-H). ^{19}F NMR (376.50 MHz, $CDCl_3$), δ: –136.5 (d, *J* = 19.2 Hz, 6F), –140.5 (d, *J* = 16.5 Hz, 2F), –151.5 (dd, *J* = 38.5, 19.2 Hz, 3F), –161.5 (t, *J* = 16.3 Hz, 6F), –161.9 (d, *J* = 13.7 Hz, 2F). MALDI-MS: *m/z* found 971.12 $[M]^+$, calcd. for $C_{44}H_{12}F_{19}N_5$ 971.08.

Synthesis of 5-[4-amino-2,3,5,6-tetrafluorophenyl]-10,15,20-tris{[4-(*m*-carboran-9'-yl)thio]-2,3,5,6-tetrafluorophenyl}porphyrin (5)

Method A. To a solution of aminoporphyrin **3** (150 mg, 0.155 mmol) in DMF (10 mL) mercaptocarborane **4** (165 mg, 0.94 mmol) and anhydrous NaOAc (38 mg, 0.46 mmol) were added. The reaction mixture was stirred for 1 h under argon at room temperature. After completion the reaction (TLC control, hexane–$CHCl_3$ 1:1) the reaction mixture was treated with water (100 mL) and extracted with $CHCl_3$ (3 × 20 mL). The obtained organic solution was dried over Na_2SO_4, the solvent was removed in vacuo, the residue was purified by column chromatography on SiO_2 using CH_2Cl_2 as an eluent to yield boronated porphyrin **5** (198 mg, 89%). IR (KBr) ν_{max}, cm^{-1}: 3324 (NH_2), 3064 (carborane CH), 2607 (BH), 1467 (CF). UV-vis (CH_2Cl_2): λ_{max} [nm] (log*ε*) = 417 (5.31), 509 (4.11), 541 (3.42), 586 (3.66). ^1H NMR (400.13 MHz, Acetone-d_6), δ: –2.85 (br s, 2H, porphyrin NH), 3.91 (br s, 6H, carborane CH), 6.05 (br s, 2H, NH_2), 9.22 (br s, 6H, β-H), 9.35 (br s, 2H, β-H). ^{19}F NMR (376.50 MHz, Acetone-d_6), δ: –133.8 (dd, *J* = 24.7, 13.7 Hz, 6F), –139.7 (dd, *J* = 24.7, 13.7 Hz, 6F), –144.1 (d, *J* = 16.5 Hz, 2F), –164.0 (d, *J* = 16.5 Hz, 2F). ^{11}B NMR (128.38 MHz, Acetone-d_6), δ: –0.9 (br s, 3B, B^9), –6.3 (d, *J* = 152 Hz, 6B), –9.7 (d, *J* = 156 Hz, 3B), –12.47 (d, *J* = 135 Hz, 6B), –13.6 (d, *J* = 163 Hz, 6B), –16.9 (d, *J* = 189 Hz, 3B). MALDI-MS: *m/z* found 1440.56 $[M]^+$, calcd. for $C_{50}H_{45}B_{30}F_{16}N_5S_3$ 1440.43.

Method B. To a solution of azidoporphyrin **2** (50 mg, 0.05 mmol) in DMF (5 mL) mercaptocarborane **4** (53 mg, 0.3 mmol) and anhydrous NaOAc (8 mg, 0.01 mmol) were added. The reaction mixture was stirred for 15 h under argon at 45 °C. After completion of the reaction the treatment as described for aminoporphyrin **3** (procedure a) was applied. The residue was purified by column chromatography on silica gel using hexane–CH_2Cl_2 1:1 to give 23 mg (32%) of boronated porphyrin **5**.

5, 10, 15-Tris{[4-(*m*-carboran-9'-yl)thio]-2,3,5,6-tetrafluorophenyl}-20-(2,3,4,5,6-pentafluorophenyl]porphyrin (6)

To a solution of porphyrin **1** (265 mg, 0.27 mmol) in DMSO (10 mL) mercaptocarborane **4** (190 mg, 1.08 mmol) and anhydrous NaOAc (67 mg, 0.82 mmol) were added and the reaction mixture was stirred for 1 h under argon at ambient temperature. Then, the mixture was treated with water (100 mL) and extracted with CH_2Cl_2 (3 × 30 mL). The combined organic solution was dried over Na_2SO_4. After removal of the solvent in vacuo, the residue was purified by column chromatography on SiO_2 using a hexane–CH_2Cl_2 1:1 as an eluent to afford 153 mg (39%) of boronated porphyrin **6** as purple solid. IR (KBr) ν_{max}, cm^{-1}: 3435 (NH), 3065 (carborane CH), 2608 (BH), 1497 (CF). UV-vis (CH_2Cl_2 λ_{max} [nm] (logε) = 415 (5.26), 508 (4.13), 586 (3.68), 649 (3.19). ^1H NMR (400.13 MHz, Acetone-d_6), δ: –2.86 (br s, 2H, porphyrin NH), 3.84 (br s, 6H, carborane CH), 9.27 (br s, 6H, β-H), 9.39 (br s, 2H, β-H). ^{19}F NMR (376.50 MHz, Acetone-d_6), δ: –133.7 (dd, *J* = 24.7, 13.7 Hz, 6F), –139.6 (dd, *J* = 27.5, 13.7 Hz, 6F), –139.8 (dd, *J* = 22.0, 5.5 Hz, 2F), –155.4 (t, *J* = 22.0 Hz, 1F), –164.4 (td, *J* = 22.0, 13.7 Hz, 2F). ^{11}B NMR (128.38 MHz, Acetone-d_6), δ: –0.9 (br s, 3B, B^9), –6.2 (d, *J* = 147 Hz, 6B), –9.7 (d, *J* = 142 Hz, 3B), –13.4 (m, 15B), –16.9 (d, *J* = 192 Hz, 3B). MALDI-MS: *m/z* found 1442.62 $[M]^+$, calcd. for $C_{50}H_{43}B_{30}F_{17}N_4S_3$ 1442.54.

5-[4-Azido-2,3,5,6-tetrafluorophenyl]-10,15,20-tris{[4-(*m*-carboran-9'-yl)thio]-2,3,5,6-tetrafluorophenyl}porphyrin (7)

A mixture of porphyrin **6** (100 mg, 0.069 mmol), sodium azide (68 mg, 1.05 mmol) in DMSO (5 mL) was stirred at room temperature under argon for 48 h under argon until the completion of the reaction (monitored by TLC). Then the reaction mixture was treated with water (100 mL) and extracted with CH_2Cl_2 (3 × 20 mL). The combined organic extracts were dried over Na_2SO_4, the solvent was removed under reduced pressure, and the residue was purified by column chromatography on silica gel using hexane–$CHCl_3$ 1:1 as an eluent to give porphyrin **7** 67 mg (66%) as violet solid. IR (KBr) ν_{max}, cm^{-1}: 3435 (NH), 3065 (carborane CH), 2608 (BH), 2127 (N_3), 1467 (CF). UV-vis (CH_2Cl_2): λ_{max} [nm] (logε) = 415

(5.52), 508 (4.35), 585 (3.96). ^{1}H NMR (300.13 MHz, Acetone-d_6), δ: –2.86 (s, 2H, porphyrin NH), 3.86 (br s, 6H, carborane CH), 9.26 (br s, 6H, β-H), 9.35 (br s, 2H, β-H). ^{19}F NMR (282.40 MHz, Acetone-d_6), δ: –133.7 (dd, *J* = 25.2, 13.8 Hz, 6F), –139.6 (dd, *J* = 25.2, 13.8 Hz, 6F), –140.7 (dd, *J* = 21.8, 12.6 Hz, 2F), –153.84 (dd, *J* = 21.8, 12.6 Hz, 2F). ^{11}B NMR (128.38 MHz, Acetone-d_6), δ: –0.97 (br s, 3B, B^9), –6.2 (d, *J* = 142 Hz, 6B), –9.7 (d, *J* = 142 Hz, 3B), –12.4 (d, *J* = 151 Hz, 6B), –13.6 (d, *J* = 165 Hz, 9B), –17.0 (d, *J* = 180 Hz, 3B). MALDI-MS: *m/z* 1467.99 found $[M+H]^+$, calcd. for $C_{50}H_{44}B_{30}F_{16}N_7S_3$ 1467.55.

Synthesis of 5-[4-amino-2,3,5,6-tetrafluorophenyl]-10,15,20-tris{[4-(*m*-carboran-9'-yl)thio]-2,3,5,6-tetrafluorophenyl}porphyrin (5)

A mixture of azidoporphyrin **7** (50 mg, 0.034 mmol) and $SnCl_2{\cdot}2H_2O$ (31 mg, 0.138 mmol) in dry MeOH (5 mL) was stirred at room temperature under argon for 30 min. After completion of the reaction (TLC control) MeOH was evaporated and the residue was purified by column chromatography on silica gel, using hexane–CH_2Cl_2 4:6 as an eluent to give 45 mg (92%) of aminoporphyrin **5**.

5,10,15-Tris{[(*m*-carboran-9'-yl)thio]-2,3,5,6-tetrafluorophenyl}-20-{[4-(2',5'-dioxo-2',5'-dihydro-1*H*-pyrrol-1'-yl)benzamido]-2,3,5,6-tetrafluorophenyl}porphyrin (11)

A mixture of *N*-(4-carboxyphenyl)maleimide (**9**, 30 mg, 0.138 mmol) and oxalyl chloride (0.5 mL) was stirred at room temperature under argon for 2 h until compete dissolution of **9**. The excess of oxalyl chloride was removed in vacuum to give the corresponding acyl chloride **8** as a white solid. Then, compound **8** was dissolved in CH_2Cl_2 (2 mL) and a solution of porphyrin **5** (66 mg, 0.0458 mmol) and Et_3N (40 μL, 0.288 mmol) in CH_2Cl_2 (5 mL) was added dropwise at 5 °C under argon for 30 min until the completion of the reaction (monitored by TLC). The reaction mixture was treated with water (100 mL) and extracted with CH_2Cl_2 (3 × 10 mL). The combined organic solution was dried over Na_2SO_4. After the removal of the solvent the product was purified by column chromatography on silica gel using CH_2Cl_2 as an eluent to give 47 mg of porphyrin **11** in 63% yield as violet solid. IR (KBr) ν_{max}, cm^{-1}: 3435 (NH), 3066 (carborane CH), 2609 (BH), 1797, 1723 (C=O), 1607 (maleimide C=C), 1467 (CF). UV-vis (CH_2Cl_2): λ_{max} [nm] (log*ε*) = 415 (5.46), 508 (4.29), 586 (3.87). ^{1}H NMR (300.13 MHz, Acetone-d_6), δ: –2.84 (br s, 2H, porphyrin NH), 3.90 (br s, 6H, carborane CH), 7.13 (s, 2H, maleimide CH=CH), 7.72 (d, *J* = 8.3 Hz, 2H, Ph), 8.35 (d, *J* = 8.5 Hz, 2H, Ph), 9.25 (br s, 6H, β-H), 9.38 (br s, 2H, β-H), 10.28 (s, 1H, NHC=O). ^{19}F NMR (282.40 MHz, Acetone-d_6), δ: –133.7 (dd, *J* = 25.2, 14.9 Hz, 6F), –139.5 (dd, *J* = 25.2, 14.9 Hz, 6F), –141.0 (dd, *J* = 23.0, 13.6 Hz, 2F), –146.3 (dd, *J* = 23.0, 13.7 Hz, 2F). ^{11}B NMR

(128.38 MHz, Acetone-d_6), δ: –0.9 (br s, 3B, B^9), –6.4 (d, J = 142 Hz, 6B), –9.6 (d, J = 132 Hz, 3B), –12.3 (d, J = 165 Hz, 6B), –13.6 (d, J = 161 Hz, 9B), –16.9 (d, J = 180 Hz, 3B). MALDI-MS: *m/z* found 1640.36 $[M]^+$, calcd. for $C_{61}H_{50}B_{30}F_{16}N_6O_3S_3$ 1639.59.

5,10,15-Tris{[4,4,4-(*m*-carboran-9'-yl)thio]-2,3,5,6-tetrafluorophenyl}-20-[4-(2'-chloroacetamido)-2,3,5,6-tetrafluorophenyl]porphyrin (12)

To a solution of porphyrin **5** (100 mg, 0.0694 mmol) in CH_2Cl_2 (8 mL) chloroacetyl chloride **10** (17 mg, 0.214 mmol) and Et_3N (30 μl, 0.216 mmol) were added at room temperature under argon, and the reaction mixture was stirred for 1 h at ambient temperature. After completion of the reaction (TLC control) the reaction mixture was treated with water (150 mL) and extracted with CH_2Cl_2 (2 × 20 mL). The obtained organic solution was dried over Na_2SO_4, the solvent was removed in vacuo, the residue was purified by column chromatography on SiO_2 using hexane–CH_2Cl_2 2:8 as an eluent to give 90 mg (85%) of acylated porphyrin **12**. IR (KBr) ν_{max}, cm^{-1}: 3468 (NH), 3066 (carborane CH), 2609 (BH), 1716 (C=O), 1468 (CF). UV-vis (CH_2Cl_2): λ_{max} [nm] (logε) = 415 (5.47), 507 (4.33), 586 (3.89). ^{1}H NMR (300.13 MHz, Acetone-d_6), δ: –2.83 (s, 2H, porphyrin NH), 3.82 (br s, 6H, carborane CH), 4.62 (br s, 2H, CH_2), 9.27 (br s, 6H, β-H), 9.37 (d, J = 3.6 Hz, 2H, β-H), 10.00 (s, 1H, NHC=O). ^{19}F NMR (282.40 MHz, Acetone-d_6), δ: –133.6 (dd, J = 25.2, 13.8 Hz, 6F), –139.5 (dd, J = 25.2, 13.8 Hz, 6F), –140.8 (dd, J = 22.9, 12.6 Hz, 2F), –146.4 (dd, J = 22.9, 12.6 Hz, 2F). ^{11}B NMR (128.38 MHz, Acetone-d_6), δ: –0.9 (br s, 3B, B^9), –6.3 (d, J = 156 Hz, 6B), –9.7 (d, J = 154 Hz, 3B), –12.4 (d, J = 147 Hz, 6B), –13.6 (d, J = 161 Hz, 9B), –17.0 (d, J = 170 Hz, 3B). MALDI-MS: *m/z* found 1517.96 $[M+H]^+$, calcd. for $C_{52}H_{47}B_{30}ClF_{16}N_5OS_3$ 1517.91.

5,10,15-Tris{[4,4,4-(*m*-carboran-9'-yl)thio]-2,3,5,6-tetrafluorophenyl}-20-[4-(2'-(4'-(4'-carboxybutyl)-2-oxohexahydro-*1H*-thieno[3,4-d]imidazol-1'-yl)acetamido)-2,3,5,6-tetrafluorophenyl]porphyrin (14)

To a solution of porphyrin **12** (60 mg, 0.0391 mmol) in dry pyridine (5 mL) biotin (**13**, 15 mg, 0.0669 mmol), Et_3N (10 μL, 0.072 mmol) and DMAP (2 mg, 0.0164mmol) were added at room temperature under argon, and the reaction mixture was stirred overnight under argon at ambient temperature. After completion of the reaction (TLC control) the solvents were removed in vacuo, and the residue was dissolved in CH_2Cl_2 (40 mL) and washed with water (3 × 50 mL). The organic solution was dried over Na_2SO_4, filtered and the residue was purified by column chromatography on SiO_2 using CH_2Cl_2–MeOH 9:1 as an eluent to give 51 mg of biotin-substituted porphyrin **14** in 76% yield. IR (KBr) ν_{max}, cm^{-1}: 3468 (OH and

NH), 3069 (carborane CH), 2609 (BH), 1715, 1641 (C=O), 1468 (CF). UV-vis ($(CH_3)_2CO$): λ_{max} [nm] (logε) = 412 (5.33), 505 (4.47), 585 (4.06). ^{1}H NMR (300.13 MHz, DMSO-d_6), δ: –3.16 (br s, 2H, porphyrin NH), 1.08 (t, J = 6.2 Hz, 4H, $(CH_2)_2$), 2.07 (d, J = 9.6 Hz, 2H, CH_2), 2.24 (t, J = 7.1 Hz, 2H, C*H2*COOH), 2.68 (m, 1H, SCH_2), 2.90 (m, 1H, SCH_2), 3.14 (d, J = 5.7 Hz, 1H, SCH), 4.14 (br s, 6H, carborane CH), 4.36 (m, 1H, CH), 4.47 (m, 1H, CH), 6.17 (br s, 2H, CH_2), 7.84 (t, J = 6.4 Hz, 1H, biotin NH), 8.80 (br s, 1H, NHC=O), 9.17 (br s, 2H, β-H), 9.22 (br s, 2H, β-H), 9.30 (d, J = 6.0 Hz, 2H, β-H), 9.38 (br s, 2H, β-H), 12.06 (br s, 1H, COOH). ^{19}F NMR (282.40 MHz, DMSO-d_6), δ: –132.9 (dd, J = 26.8, 11.5 Hz, 6F), –138.7 (dd, J = 26.8, 11.5 Hz, 6F), –140.3 (d, J = 18.7 Hz, 2F), –144.2 (d, J = 16.5 Hz, 2F). ^{11}B NMR (128.38 MHz, DMSO-d_6), δ: –0.9 (br s, 3B, B^9), –6.1 (d, J = 149 Hz, 6B), –9.6 (d, J = 144 Hz, 3B), –12.4 (d, J = 149 Hz, 6B), –13.6 (d, J = 161 Hz, 9B), –16.9 (d, J = 180 Hz, 3B). MALDI-MS: *m/z* found 1722.56 $[M]^+$, calcd. for $C_{62}H_{61}B_{30}F_{16}N_7O_4S_4$ 1722.65.

General procedure for preparation of boronated porphyrins 18–20

To a mixture of porphyrin **6** (52 mg, 0.036 mmol) and mercaptoethanol (**15**, 5 μL, 0.071 mmol) or cysteamine hydrochloride (**16**, 20 mg, 0.176 mmol) or 3-chloro-1-propanethiol (**17**, 7 μL, 0.072 mmol) in DMSO (5 mL) anhydrous NaOAc (6 mg, 0.073 mmol) was added and the reaction mixture was stirred for 10 min at ambient temperature under argon until the completion of the reaction (monitored by TLC). Then, the reaction mixture was treated with water (100 mL) (for porphyrin **19** also treated Et_3N (1 mL)), and extracted with CH_2Cl_2 (3 × 20 mL). The combined organic extracts were dried over Na_2SO_4, the solvent was removed under reduced pressure and the residue was purified by column chromatography on silica gel using $CHCl_3$ as an eluent (for porphyrin **18** and **19**) or hexane–$CHCl_3$ 1:1 mixture as an eluent (for porphyrin **20**) to give porphyrins **18–20** in 80–87%yields as violet solids.

5,10,15-Tris{[(*m*-carboran-9'-yl)thio]-2,3,5,6-tetrafluorophenyl}-20-{[(4-(2'-hydroxyethyl)thio]-2,3,5,6-tetrafluorophenyl}porphyrin (18)

Yield: 47 mg (87 %). IR (KBr) ν_{max}, cm^{-1}: 3435 (NH), 3065 (carborane CH), 2609 (BH), 1468 (CF). UV-vis (CH_2Cl_2): λ_{max} [nm] (logε) = 416 (5.40), 509 (4.23), 585 (3.78). ^{1}H NMR (400.13 MHz, Acetone-d_6), δ: –2.87 (s, 2H, porphyrin NH), 3.44 (t, J = 6.4 Hz, 2H, CH_2), 3.88 (br s, 6H, carborane CH), 4.02 (q, J = 5.8 Hz, 2H, CH_2), 4.27 (t, J = 5.1 Hz, 1H, OH), 9.25 (br s, 6H, β-H), 9.37 (br s, 2H, β-H). ^{19}F NMR (376.50 MHz, Acetone-d_6), δ: –133.8 (dd, J = 24.7, 13.7 Hz, 6F), –135.7 (dd, J = 24.7, 13.7 Hz, 2F), –139.7 (dd, J = 27.5, 13.7 Hz, 6F), –140.2 (dd, J = 24.7, 13.7 Hz, 2F). ^{11}B NMR (128.38 MHz, Acetone-d_6), δ: –0.9 (br s, 3B, B^9), –6.3

(d, *J* = 149 Hz, 6B), –9.6 (d, *J* = 156 Hz, 3B), –12.5 (d, *J* = 130 Hz, 6B), –13.6 (d, *J* = 161 Hz, 9B), –17.0 (d, *J* = 184 Hz, 3B). MALDI-MS: *m/z* found 1501.00 $[M]^+$, calcd. for $C_{52}H_{48}B_{30}F_{16}N_4OS_4$ 1500.55.

5-{[4-(2'-Aminoethyl)thio]-2,3,5,6-tetrafluorophenyl}-10,15,20-tris{[4,4,4-(*m*-carboran-9'-yl)thio]-2,3,5,6-tetrafluorophenyl}porphyrin (19)

Yield: 43 mg (80%). IR (KBr) ν_{max}, cm^{-1}: 3437 (NH), 3064 (carborane CH), 2609 (BH), 1468 (CF). UV-vis (CH_2Cl_2): λ_{max} [nm] (logε) = 414 (5.30), 508 (4.15), 585 (3.73). ^1H NMR (300.13 MHz, Acetone-d_6), δ: –2.87 (s, 2H, porphyrin NH), 3.54 (t, *J* = 6.5 Hz, 2H, CH_2), 3.72 (t, *J* = 6.5 Hz, 2H, CH_2), 3.89 (br s, 6H, carborane CH), 9.27 (br s, 8H, β-H). ^{19}F NMR (282.40 MHz, Acetone-d_6), δ: –133.7 (dd, *J* = 24.1, 13.8 Hz, 6F), –135.6 (dd, *J* = 25.2, 14.9 Hz, 2F), –139.6 (dd, *J* = 25.2, 12.6 Hz, 6F), –140.3 (dd, *J* = 26.4, 13.8 Hz, 2F). ^{11}B NMR (128.38 MHz, Acetone-d_6), δ: –0.9 (br s, 3B, B^9), –6.2 (d, *J* = 151 Hz, 6B), –9.7 (d, *J* = 156 Hz, 3B), –12.5 (d, *J* = 132 Hz, 6B), –13.6 (d, *J* = 163 Hz, 9B), –16.9 (d, *J* = 189 Hz, 3B). MALDI-MS: *m/z* found 1500.84 $[M+H]^+$, calcd. for $C_{52}H_{50}B_{30}F_{16}N_5S_4$ 1500.56.

5,10,15-Tris{[4,4,4-(*m*-carboran-9'-yl)thio]-2,3,5,6-tetrafluorophenyl}-20-{[(4-(3'-chloropropyl)thio]-2,3,5,6-tetrafluorophenyl]porphyrin (20)

Yield: 47 mg (85%). IR (KBr) ν_{max}, cm^{-1}: 3322 (NH), 3064 (carborane CH), 2607 (BH), 1467 (CF). UV-vis (CH_2Cl_2): λ_{max} [nm] (logε) = 415 (5.42), 508 (4.26), 541 (3.58), 585 (3.82), 657 (3.46). ^1H NMR (300.13 MHz, Acetone-d_6), δ: –2.87 (br s, 2H, porphyrin NH), 2.35 (t, *J* = 6.6 Hz, 2H, CH_2), 3.45 (t, *J* = 7.0 Hz, 2H, CH_2), 3.92 (br s, 6H, carborane CH), 3.94 (t, *J* = 6.0 Hz, 2H, CH_2), 9.25 (br s, 6H, β-H), 9.35 (br s, 2H, β-H). ^{19}F NMR (376.50 MHz, Acetone-d_6), δ: –133.7 (dd, *J* = 24.8, 13.8 Hz, 6F), –135.6 (dd, *J* = 24.8, 13.8 Hz, 2F), –139.7 (dd, *J* = 24.8, 13.8 Hz, 8F). ^{11}B NMR (128.38 MHz, Acetone-d_6), δ: –0.9 (br s, 3B, B^9), –6.3 (d, *J* = 149 Hz, 6B), –9.7 (d, *J* = 152 Hz, 3B), –12.4 (d, *J* = 147 Hz, 6B), –13.6 (d, *J* = 161 Hz, 9B), –16.9 (d, *J* = 182 Hz, 3B). MALDI-MS: *m/z* found 1534.07 $[M+H]^+$, calcd. for $C_{53}H_{50}B_{30}ClF_{16}N_4S_4$ 1533.53.

General procedure for preparation of boronated porphyrins 23, 24, 26

To a mixture of porphyrin **6** (52 mg, 0.036 mmol) and 1,8-diamino-3,6-dioxaoctane (**21**, 27 μL, 0.184 mmol) or 1,13-diamino-4,7,10-trioxatridecane (**22**, 40 μL, 0.184 mmol) or taurine (**25**, 23 mg, 0.184 mmol) in DMSO (5 mL) anhydrous NaOAc (15 mg, 0.184 mmol) was added and the reaction mixture was stirred for 30 min at 70 °C for conjugates **23** and **24** and 72 h at room temperature for conjugate **26** under argon until the completion of the

reaction (monitored by TLC). Then the reaction mixture was treated with water (100 mL) and extracted with ethyl acetate (3 × 20 mL). The combined organic extracts were dried over Na_2SO_4, the solvent was removed under reduced pressure and the residue was purified by column chromatography on silica gel using acetone as an eluent.

5-[(4-(2’-(2’-(2’-Aminoethoxy)ethoxy)ethyl)amino)-2,3,5,6-tetrafluorophenyl]-10,15,20-*tris*{[4,4,4-(*m*-carboran-9’-yl)thio]-2,3,5,6-tetrafluorophenyl}porphyrin (23)

Yield: 40 mg (71 %). IR (KBr) ν_{max}, cm^{-1}: 3429 (NH and NH_2), 3061 (carborane CH), 2605 (BH), 1466 (CF). UV-vis (CH_2Cl_2): λ_{max} [nm] (logε) = 415 (5.22), 508 (4.23), 587 (3.85), 651 (3.85). ^1H NMR (300.13 MHz, Acetone-d6), δ: –2.86 (br s, 2H, porphyrin NH), 2.97 (br s, 2H, CH_2), 3.73 (br s, 4H, CH_2), 3.86 (br s, 6H, CH_2), 3.92 (br s, 6H, carborane CH), 9.22 (br s, 6H, β-H), 9.35 (br s, 2H, β-H). ^{19}F NMR (282.40 MHz, Acetone-d6), δ: –133.8 (dd, *J* = 18.4, 6.9 Hz, 6F), –139.7 (dd, *J* = 26.4, 13.8 Hz, 6F), –143.5 (dd, *J* = 22.2, 9.2 Hz, 2F), –161.7 (dd, *J* = 25.2, 4.6 Hz, 2F). ^{11}B NMR (128.38 MHz, Acetone-d6), δ: –0.9 (br s, 3B, B^9), –6.2 (d, *J* = 135 Hz, 6B), –9.7 (d, *J* = 158 Hz, 3B), –12.4 (d, *J* = 142 Hz, 6B), –13.6 (d, *J* = 163 Hz, 9B), –17.0 (d, *J* = 182 Hz, 3B). MALDI-MS: *m/z* found 1572.44 $[M+H]^+$, calcd. for $C_{56}H_{59}B_{30}F_{16}N_6O_2S_3$ 1571.65.

5-[(4-(((3’-(2’-(2’-(3’-Aminopropoxy)ethoxy)ethoxy)propyl)amino)-2,3,5,6-tetrafluorophenyl]-10,15,20-tris{[4,4,4-(*m*-carboran-9’-yl)thio]-2,3,5,6-tetrafluorophenyl}porphyrin (24)

Yield: 50 mg (84%). IR (KBr) ν_{max}, cm^{-1}: 3430 (NH and NH_2), 3062 (carborane CH), 2606 (BH), 1466 (CF). UV-vis (CH_2Cl_2): λ_{max} [nm] (logε) = 415 (5.22), 508 (4.18), 587 (3.81). ^1H NMR (400.13 MHz, Acetone-d6), δ: –2.86 (br s, 2H, porphyrin NH), 2.97 (br s, 2H, CH_2), 3.05 (t, *J* = 6.4 Hz, 4H, $(CH_2)_2$), 3.56 (m, 10H, CH_2), 3.67 (br s, 2H, CH_2), 3.75 (br s, 2H, CH_2), 3.91 (br s, 6H, carborane CH), 9.23 (br s, 6H, β-H), 9.35 (br s, 2H, β-H). ^{19}F NMR (282.40 MHz, Acetone-d6), δ: –133.8 (dd, *J* = 25.2, 13.8 Hz, 6F), –139.7 (dd, *J* = 25.2, 13.8 Hz, 6F), –143.7 (dd, *J* = 19.7, 11.5 Hz, 2F), –161.7 (dd, *J* = 19.7, 11.5 Hz, 2F). ^{11}B NMR (128.38 MHz, Acetone-d6), δ: –0.9 (br s, 3B, B^9), –6.2 (d, *J* = 135 Hz, 6B), –9.7 (d, *J* = 154 Hz, 3B), –12.4 (d, *J* = 149 Hz, 6B), –13.6 (d, *J* = 165 Hz, 9B), –17.0 (d, *J* = 182 Hz, 3B). MALDI-MS: *m/z* found 1642.77 $[M]^+$, calcd. for $C_{60}H_{66}B_{30}F_{16}N_6O_3S_3$ 1642.71.

5,10,15 -Tris{[4,4,4-(*m*-carboran-9'-yl)thio]-2,3,5,6-tetrafluorophenyl}-20-[4-((2'-sulfoethyl)amino)-2,3,5,6-tetrafluorophenyl]porphyrin (26)

Yield: 44 mg (78%). IR (KBr) ν_{max}, cm^{-1}: 3434 (NH), 3063 (carborane CH), 2608 (BH), 1468 (CF). UV-vis (THF): λ_{max} [nm] (logε) = 416 (5.33), 508 (4.25), 586 (3.87). ^{1}H NMR (300.13 MHz, THF-d_8), δ: –2.83 (br s, 2H, porphyrin NH), 3.52 (br s, 2H, CH_2), 3.83 (br s, 6H, carborane CH), 4.32 (br s, 2H, CH_2), 6.43 (br s, 1H, NH), 9.04 (br s, 6H, β-H), 9.22 (br s, 2H, β-H). ^{19}F NMR (282.40 MHz, THF-d_8), δ: –129.8 (dd, *J* = 25.2, 13.8 Hz, 6F), –135.6 (dd, *J* = 25.2, 14.98 Hz, 6F), –139.2 (d, *J* = 17.2 Hz, 2F), –158.3 (d, *J* = 14.9 Hz, 2F). ^{11}B NMR (128.38 MHz, THF-d_8), δ: –0.9 (br s, 3B, B^9), –6.3 (d, *J* = 144 Hz, 6B), –9.7 (d, *J* = 140 Hz, 3B), –12.5 (d, *J* = 140 Hz, 6B), –13.6 (d, *J* = 163 Hz, 9B), –17.0 (d, *J* = 184 Hz, 3B). MALDI-MS: *m/z* found 1547.97 $[M]^+$, calcd. for $C_{52}H_{49}B_{30}F_{16}N_5O_3S_4$ 1547.55.

3. The ^{1}H and ^{19}F NMR spectra of 5-(4-azido-2,3,5,6-tetrafluorophenyl)-10,15,20-tris(pentafluorophenyl)porphyrin (2)

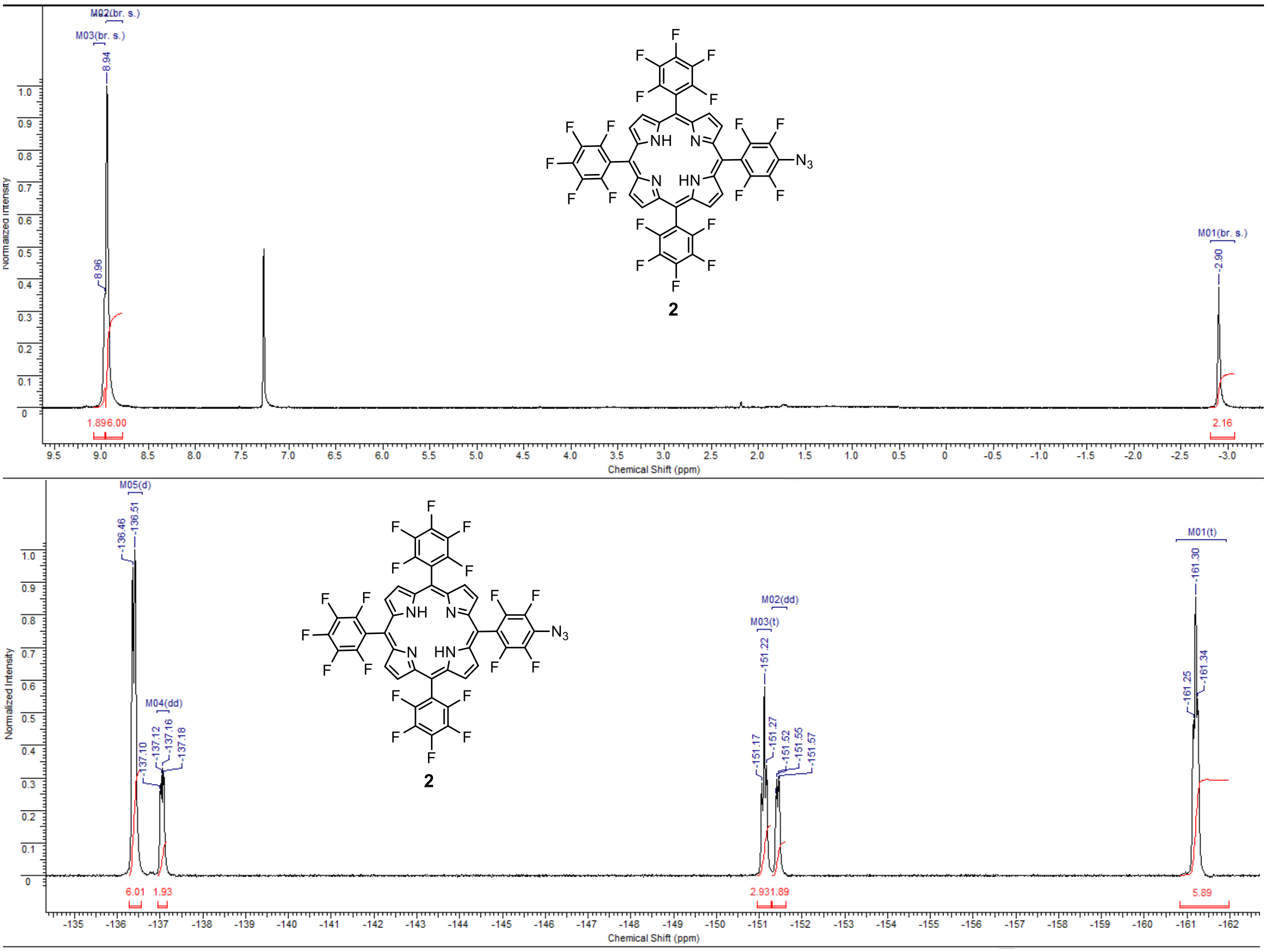

Figure S1. The ^{1}H, ^{19}F NMR spectra of **2** in $CDCl_3$.

4. The ^{1}H and ^{19}F NMR spectra of 5-(4-amino-2,3,5,6-tetrafluorophenyl)-10,15,20-tris(pentafluorophenyl)porphyrin (3)

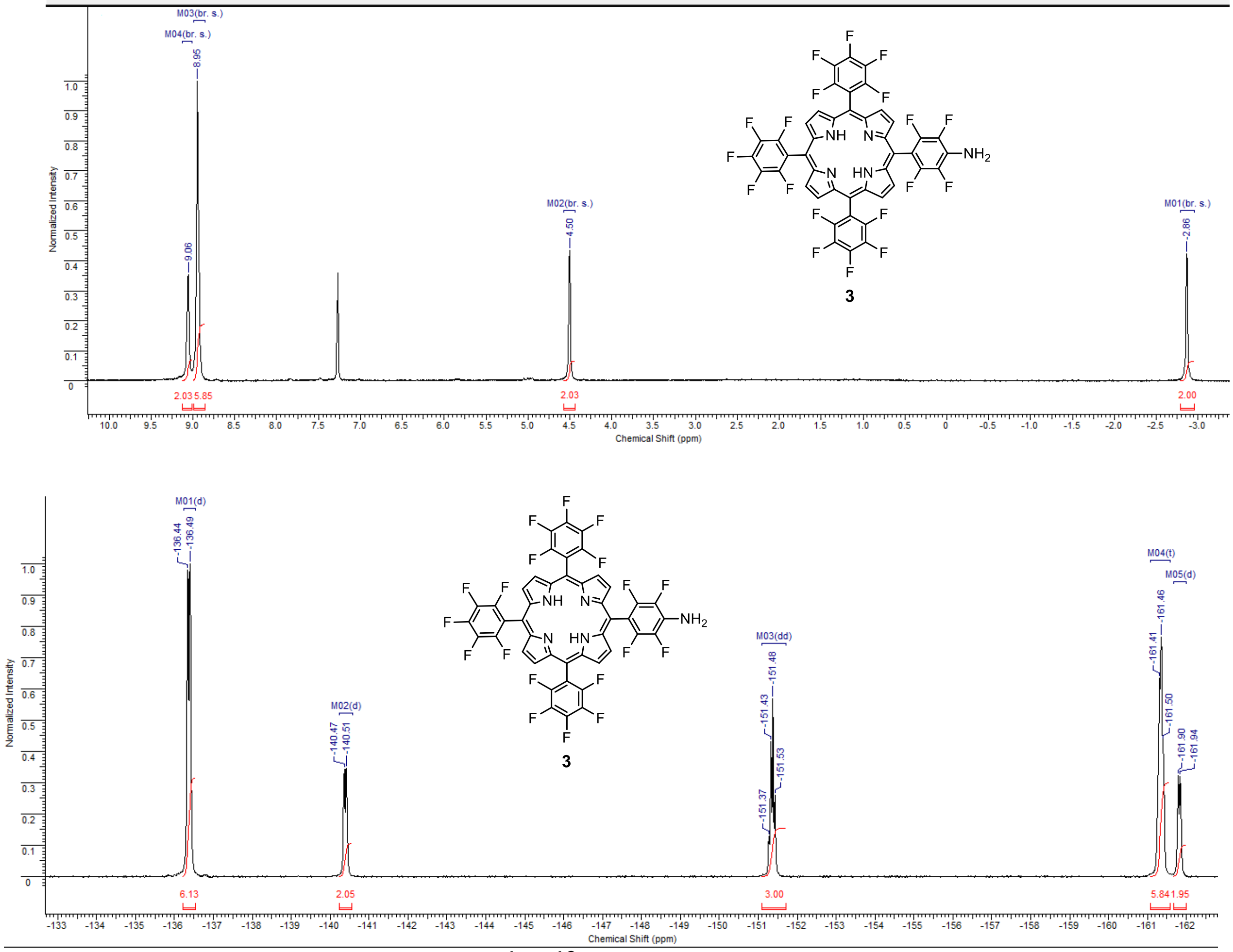

Figure S2.The ^{1}H, ^{19}F NMR spectra of **3** in $CDCl_3$.

5. The ^{1}H, ^{19}F and ^{11}B NMR spectra of 5-[4-amino-2,3,5,6-tetrafluorophenyl]-10,15,20-tris{[4-(*m*-carboran-9'-yl)thio]-2,3,5,6-tetrafluorophenyl}porphyrin (5)

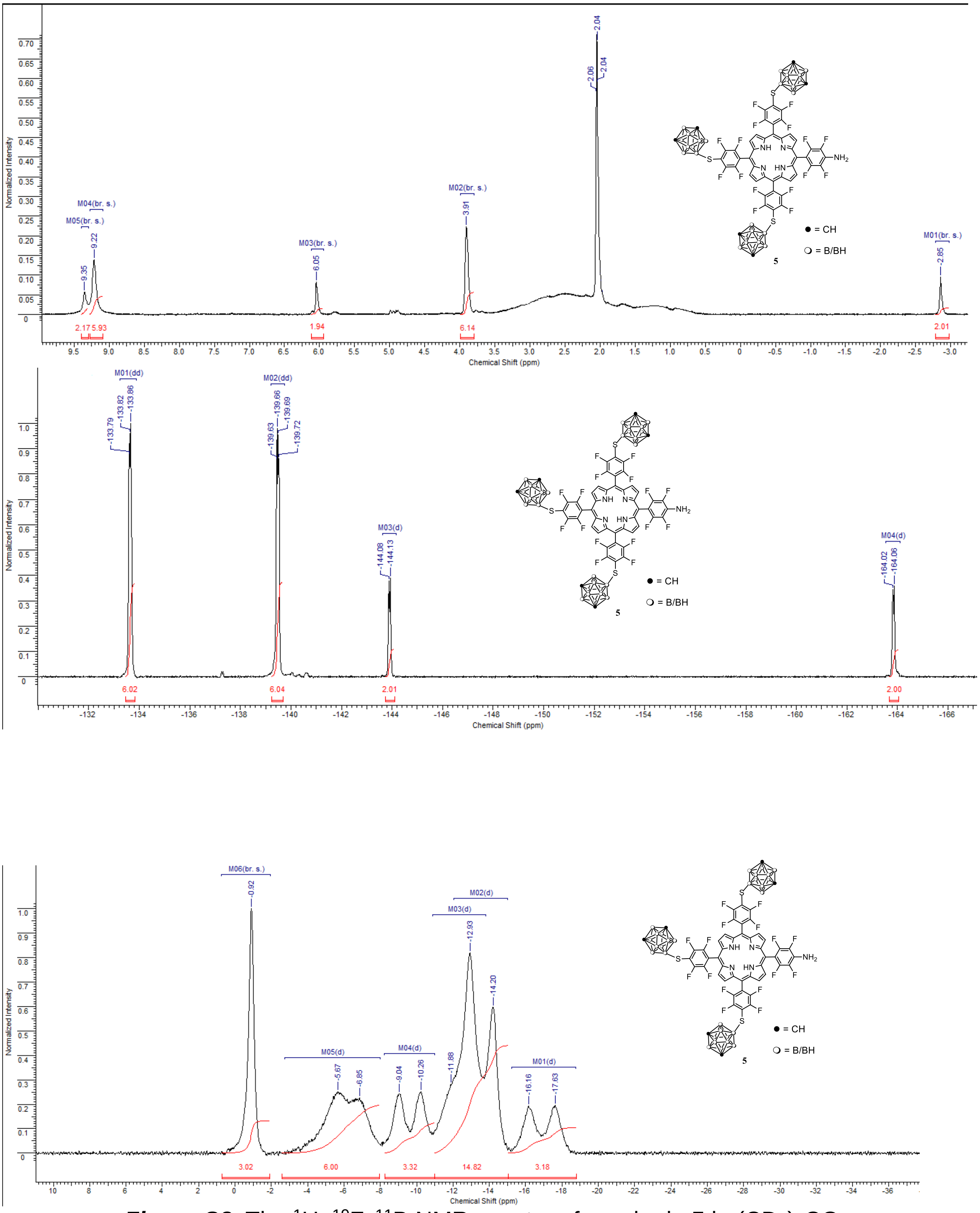

Figure S3. The ^{1}H, ^{19}F, ^{11}B NMR spectra of porphyrin **5** in $(CD_3)_2CO$.

6. The ^{1}H, ^{19}F and ^{11}B NMR spectra of 5, 10, 15-tris{[4-(*m*-carboran-9'-yl)thio]-2,3,5,6-tetrafluorophenyl}-20-(2,3,4,5,6-pentafluorophenyl]porphyrin (6)

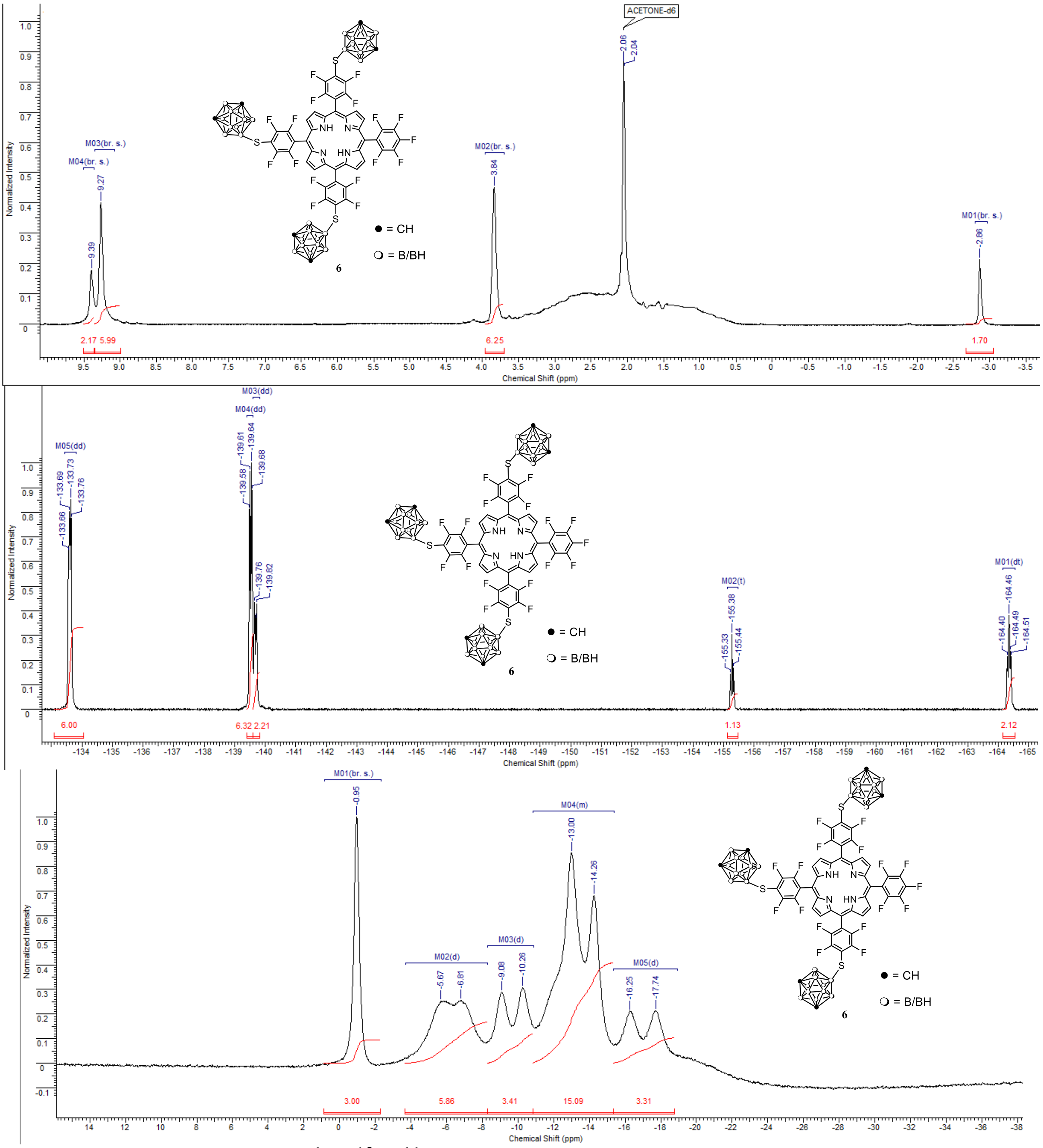

Figure S4. The ^{1}H, ^{19}F, ^{11}B NMR spectra of porphyrin **6** in $(CD_3)_2CO$.

7. The ^{1}H, ^{19}F and ^{11}B NMR spectra of 5-[4-azido-2,3,5,6-tetrafluorophenyl]-10,15,20-tris{[4-(*m*-carboran-9’-yl)thio]-2,3,5,6-tetrafluorophenyl}porphyrin (7)

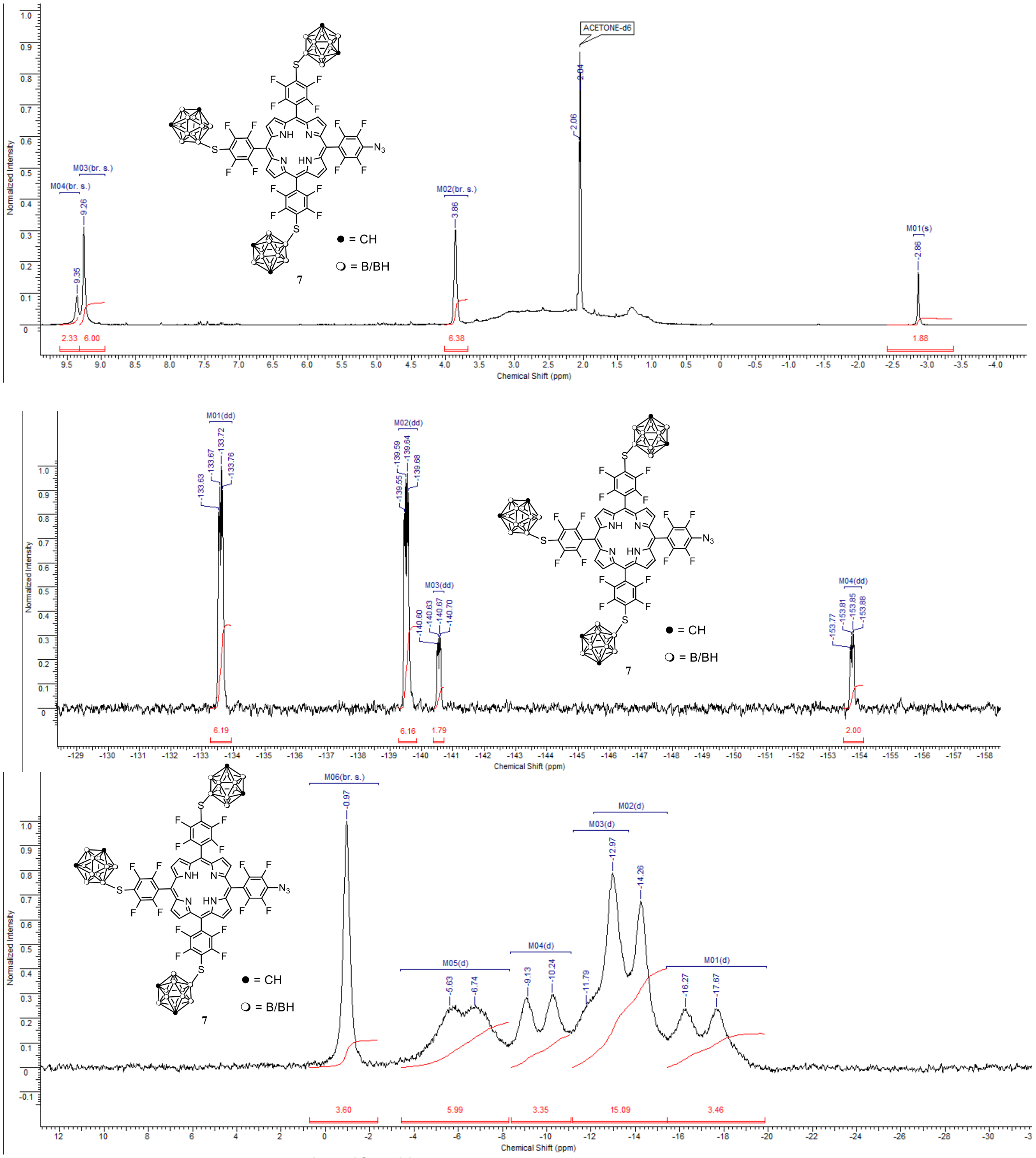

Figure S5. The ^{1}H, ^{19}F, ^{11}B NMR spectra of porphyrin **7** in $(CD_3)_2CO$.

8. The ^{1}H, ^{19}F and ^{11}B NMR spectra of 5,10,15-tris{[(*m*-carboran-9'-yl)thio]-2,3,5,6-tetrafluorophenyl}-20-{[4-(2',5'-dioxo-2',5'-dihydro-1*H*-pyrrol-1'-yl)benzamido]-2,3,5,6-tetrafluorophenyl}porphyrin (11)

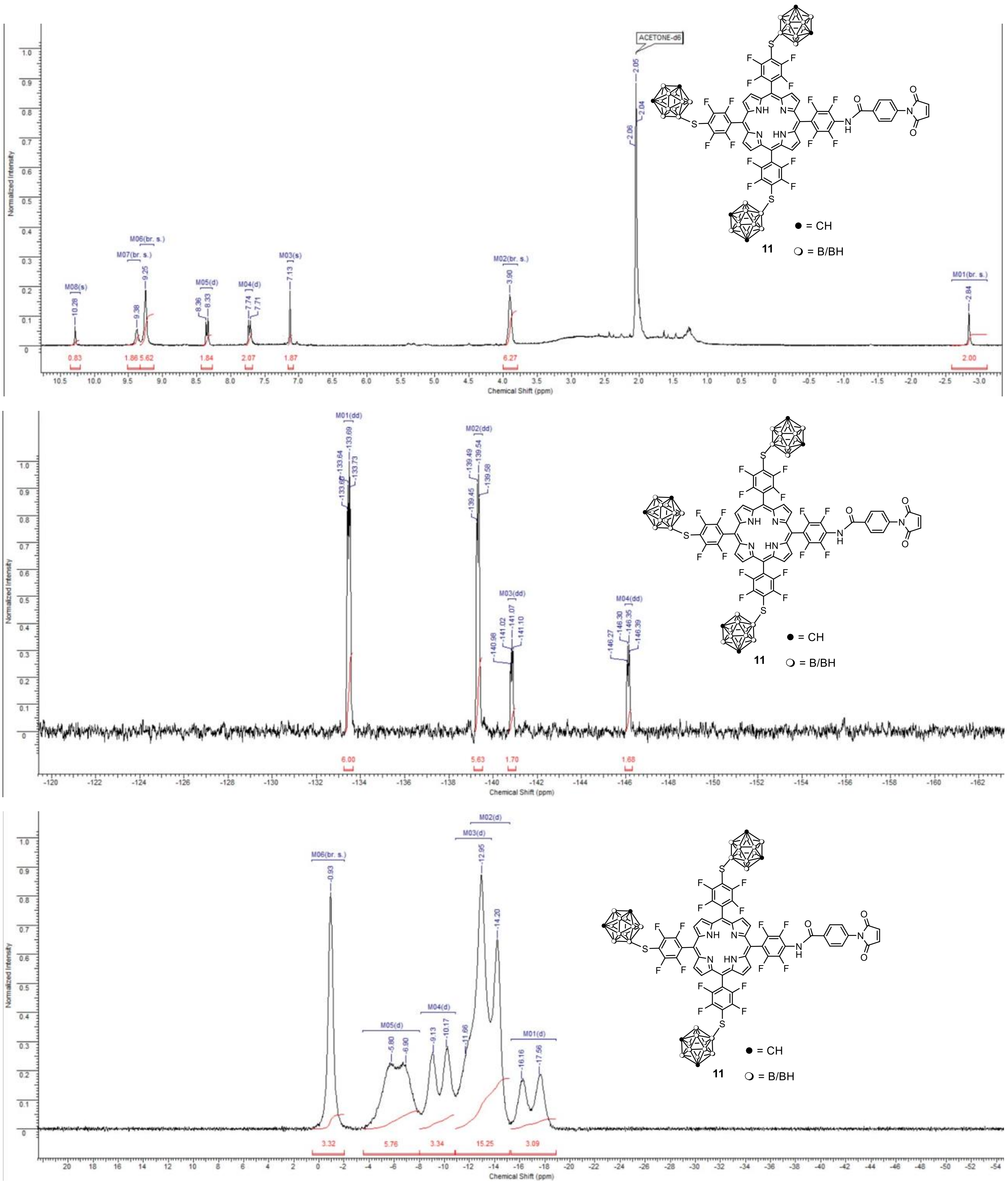

Figure S6. The ^{1}H, ^{19}F, ^{11}B NMR spectra of porphyrin **11** in $(CD_3)_2CO$.

9. The ^{1}H, ^{19}F and ^{11}B NMR spectra of 5,10,15-tris{[4,4,4-(*m*-carboran-9'-yl)thio]-2,3,5,6-tetrafluorophenyl}-20-[4-(2'-chloroacetamido)-2,3,5,6-tetrafluorophenyl]porphyrin (12)

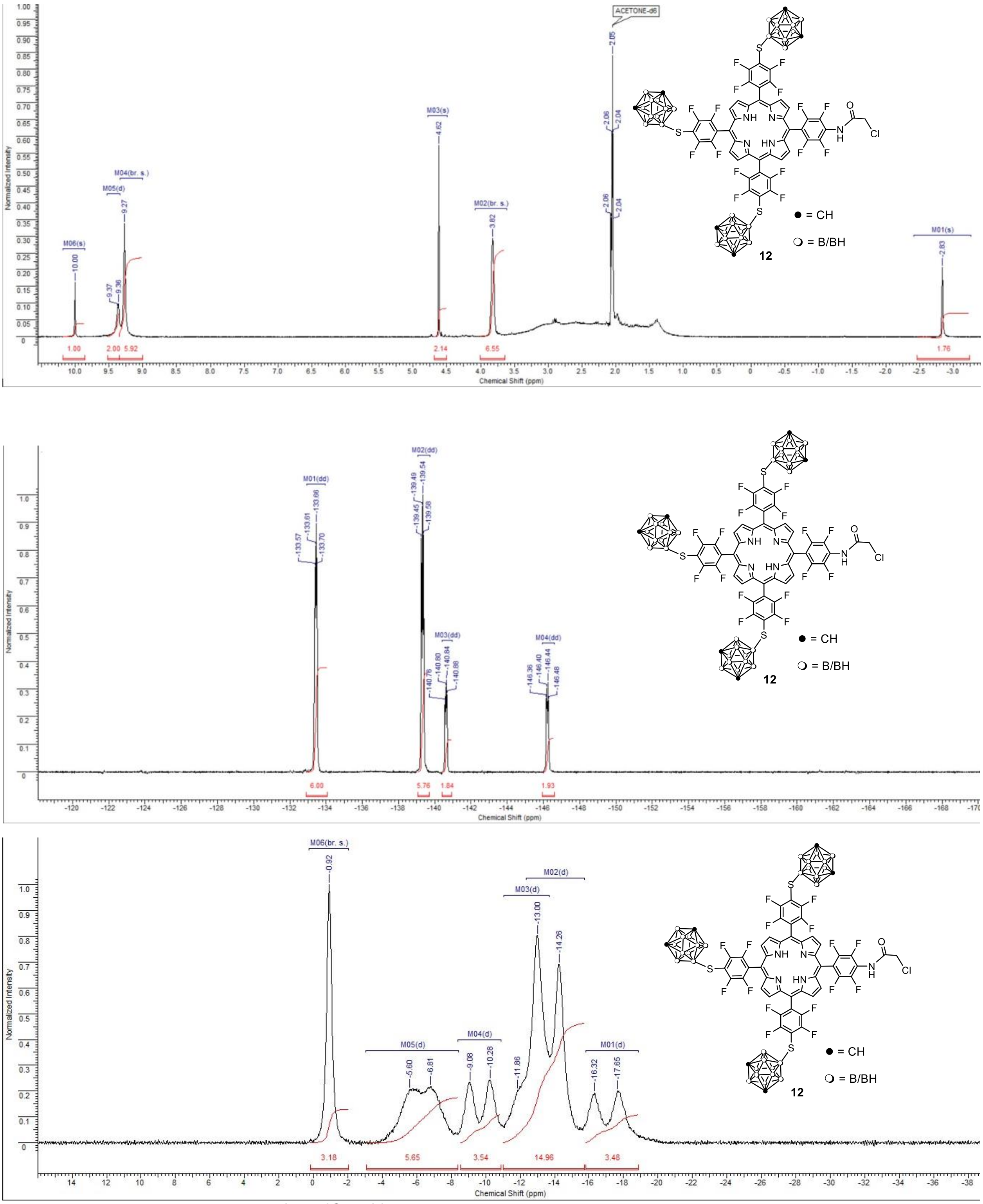

Figure S7. The ^{1}H, ^{19}F, ^{11}B NMR spectra of porphyrin **12** in $(CD_3)_2CO$.

10. The ^{1}H, ^{19}F and ^{11}B NMR spectra of 5,10,15-tris{[4,4,4-(*m*-carboran-9'-yl)thio]-2,3,5,6-tetrafluorophenyl}-20-[4-(2'-(4'-(4'-carboxybutyl)-2-oxohexahydro-*1H*-thieno[3,4-d]imidazol-1'-yl)acetamido)-2,3,5,6-tetrafluorophenyl]porphyrin (14)

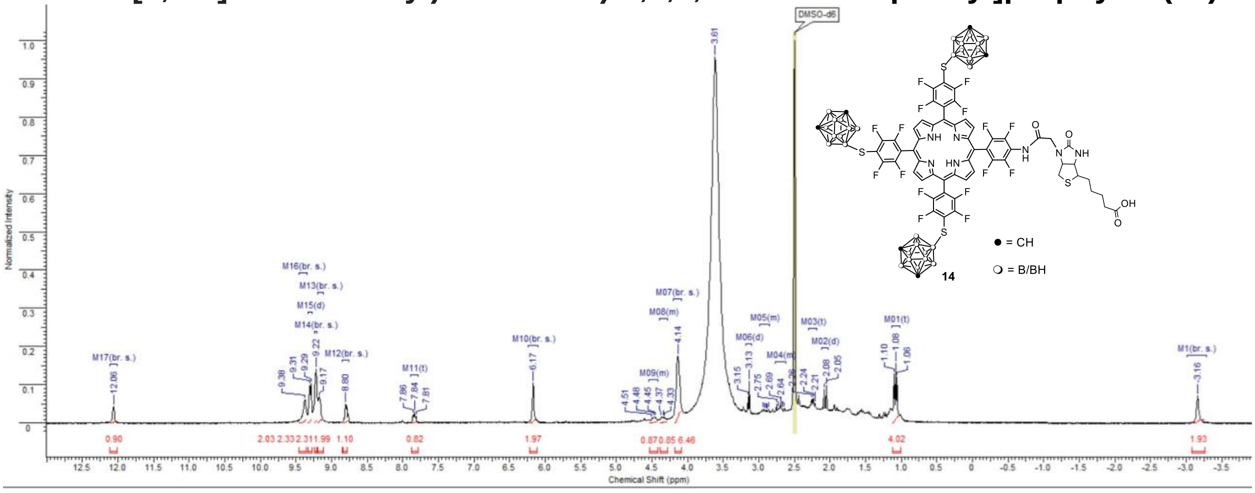

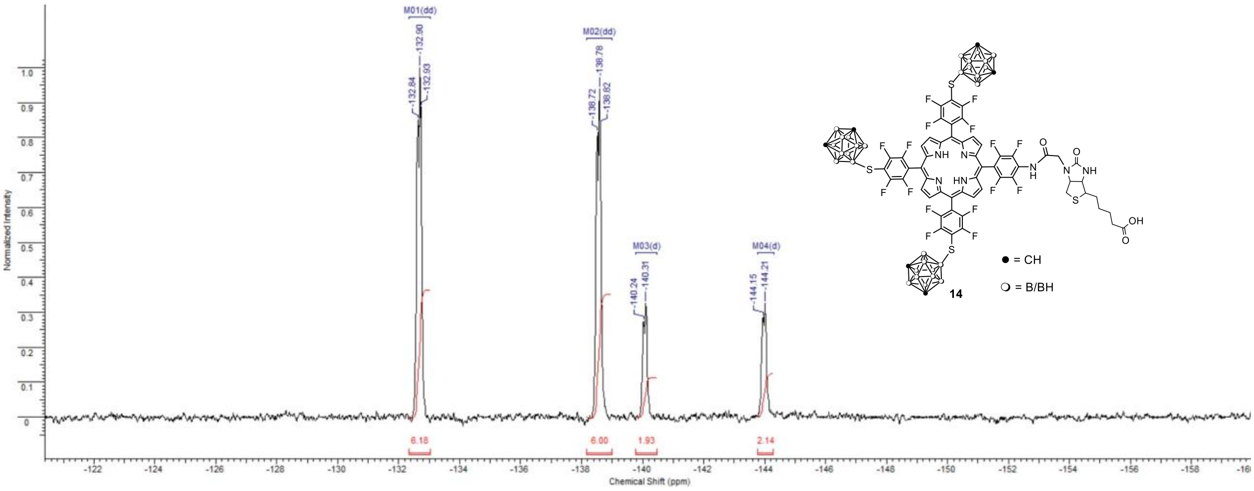

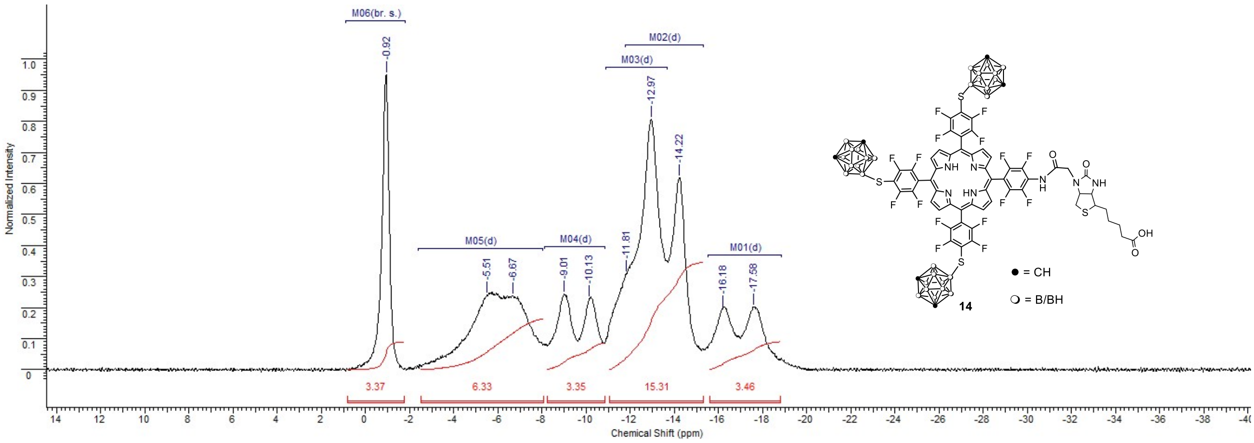

Figure S8. The ^{1}H, ^{19}F, ^{11}B NMR spectra of porphyrin **14** in $(CD_3)_2SO$.

11. The ^{1}H, ^{19}F and ^{11}B spectra of 5,10,15-tris{[(*m*-carboran-9'-yl)thio]-2,3,5,6-tetrafluorophenyl}-20-{[(4-(2'-hydroxyethyl)thio]-2,3,5,6-tetrafluorophenyl}porphyrin (18)

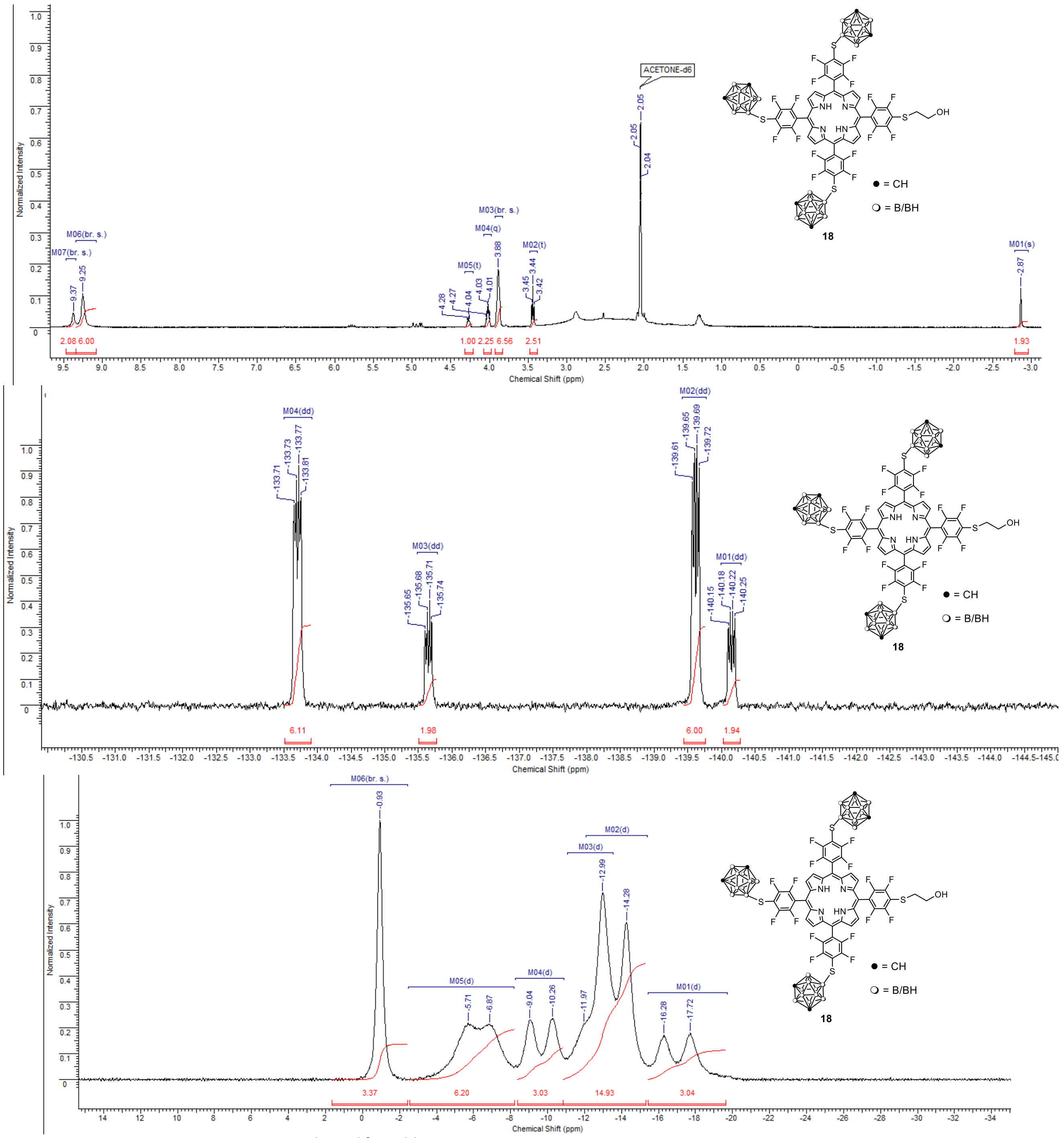

Figure S9. The ^{1}H, ^{19}F, ^{11}B NMR spectra of porphyrin **18** in $(CD_3)_2CO$.

12. The ^{1}H, ^{19}F and ^{11}B spectra of 5-{[4-(2'-aminoethyl)thio]-2,3,5,6-tetrafluorophenyl}-10,15,20-tris{[4,4,4-(*m*-carboran-9'-yl)thio]-2,3,5,6-tetrafluorophenyl}porphyrin (19)

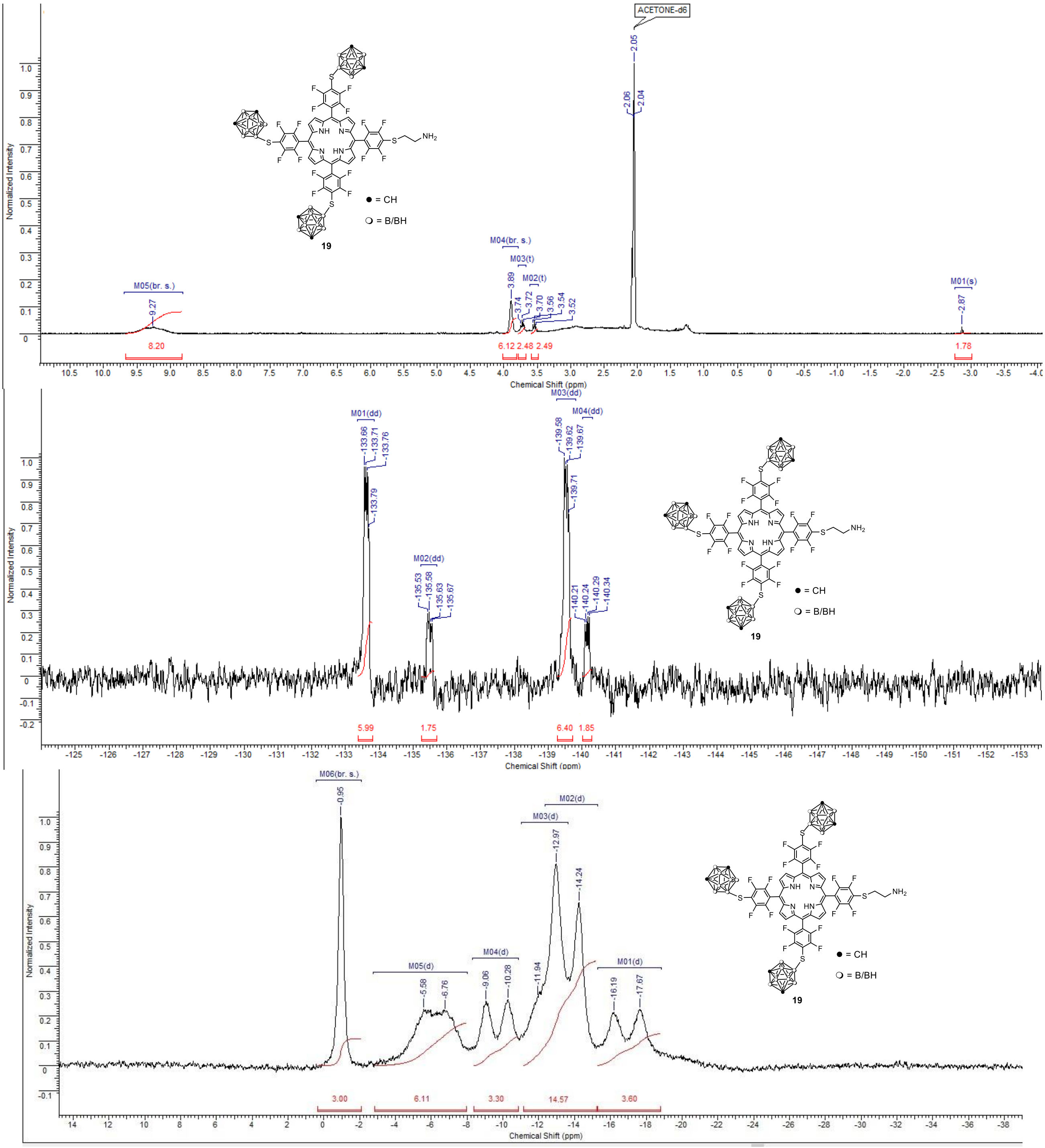

Figure S10. The ^{1}H, ^{19}F, ^{11}B NMR spectra of porphyrin **19** in $(CD_3)_2CO$.

13. The ^{1}H, ^{19}F and ^{11}B spectra of 5,10,15 -tris{[4,4,4-(*m*-carboran-9'-yl)thio]-2,3,5,6-tetrafluorophenyl}-20-{[(4-(3'-chloropropyl)thio]-2,3,5,6-tetrafluorophenyl]porphyrin (20)

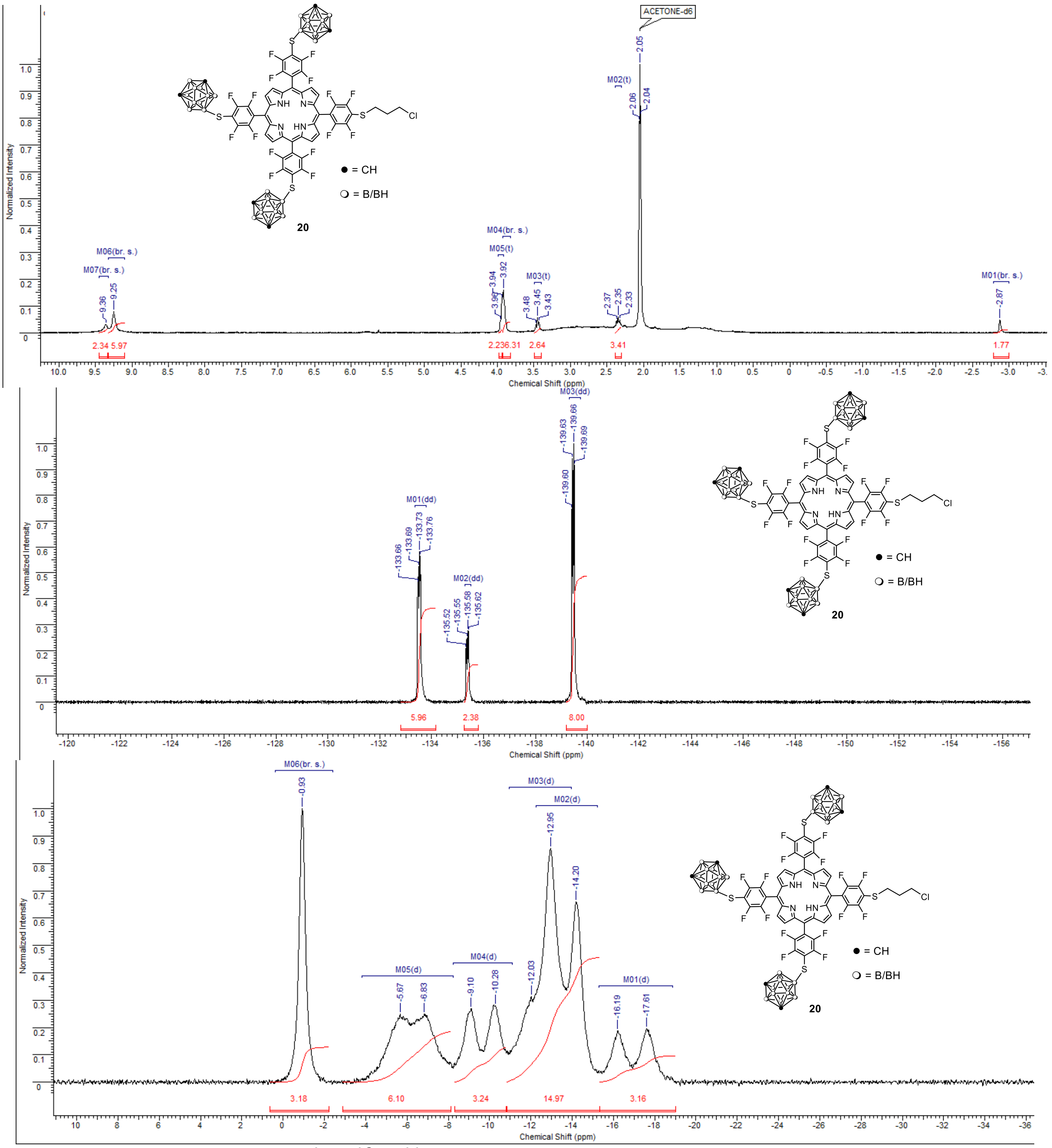

Figure S11. The ^{1}H, ^{19}F, ^{11}B NMR spectra of porphyrin **20** in $(CD_3)_2CO$.

14. The ^{1}H, ^{19}F and ^{11}B spectra of 5-[(4-(2’-(2’-(2’-aminoethoxy)ethoxy)ethyl)amino)-2,3,5,6-tetrafluorophenyl]-10,15,20-tris{[4,4,4-(*m*-carboran-9’-yl)thio]-2,3,5,6-tetrafluorophenyl}porphyrin (23)

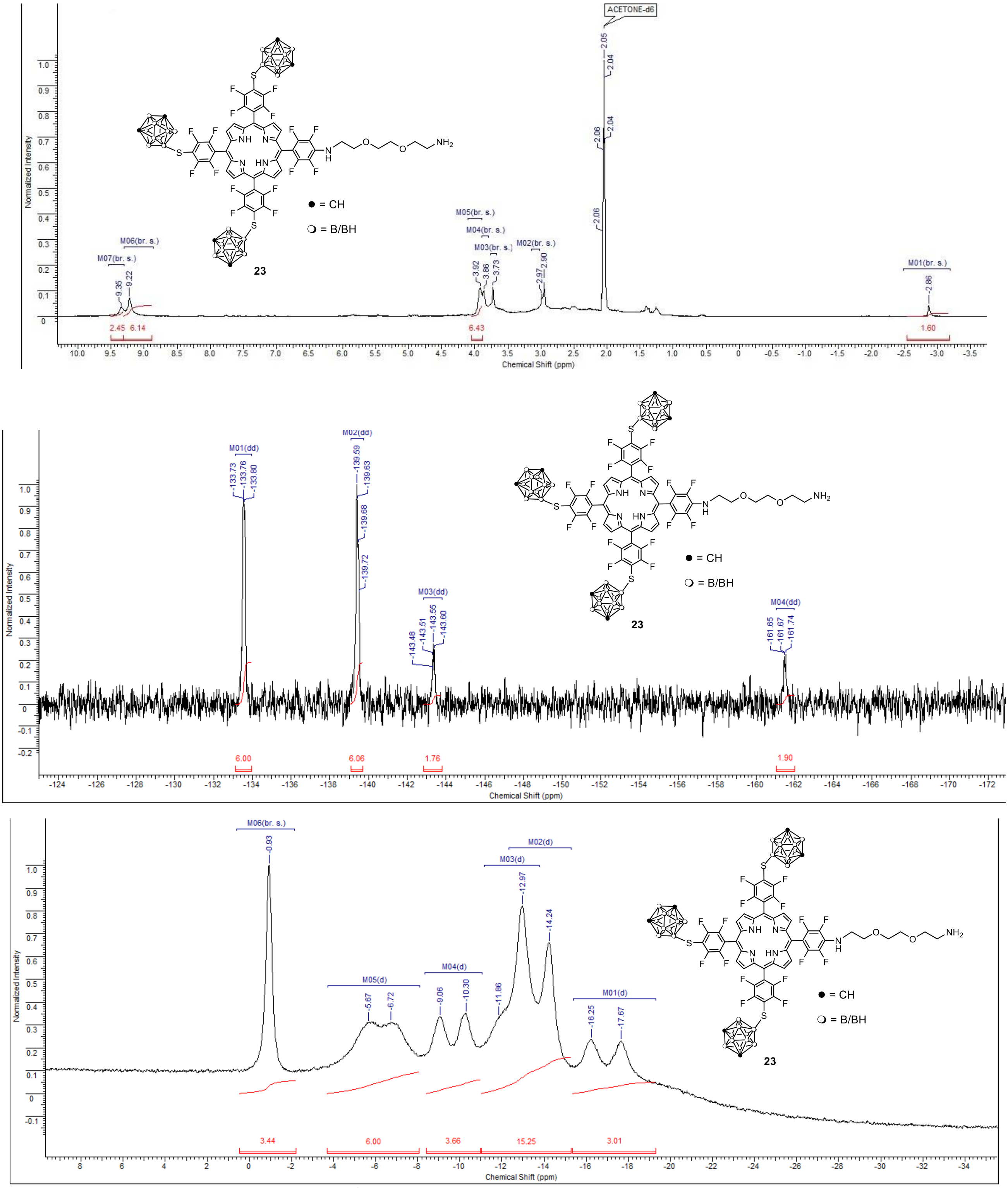

Figure S12. The ^{1}H, ^{19}F, ^{11}B NMR spectra of porphyrin **23** in $(CD_3)_2CO$.

15. The ^{1}H, ^{19}F and ^{11}B spectra of 5-[(4-(((3’-(2’-(2’-(3’-aminopropoxy)ethoxy)ethoxy)propyl)amino)-2,3,5,6-tetrafluorophenyl]-10,15,20-tris{[4,4,4-(*m*-carboran-9’-yl)thio]-2,3,5,6-tetrafluorophenyl}porphyrin (24)

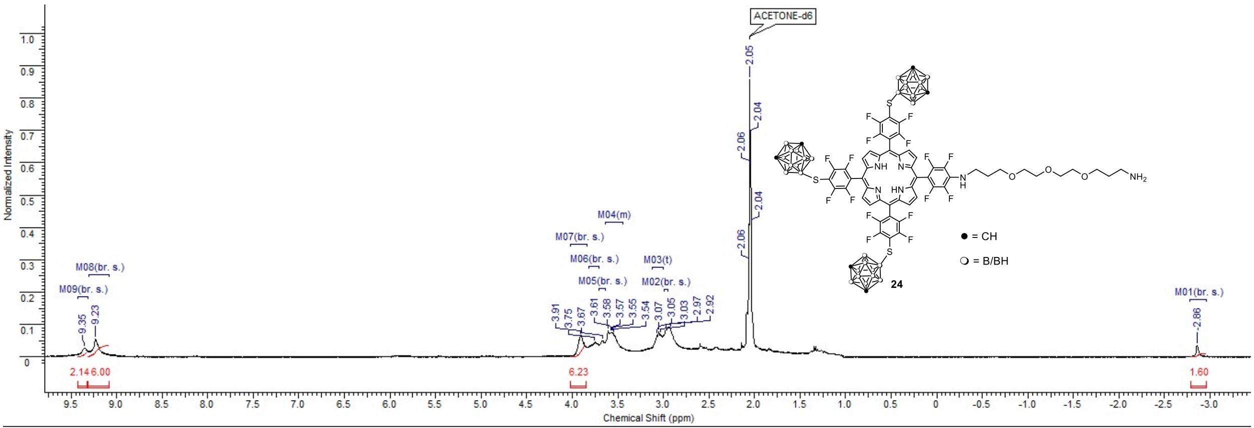

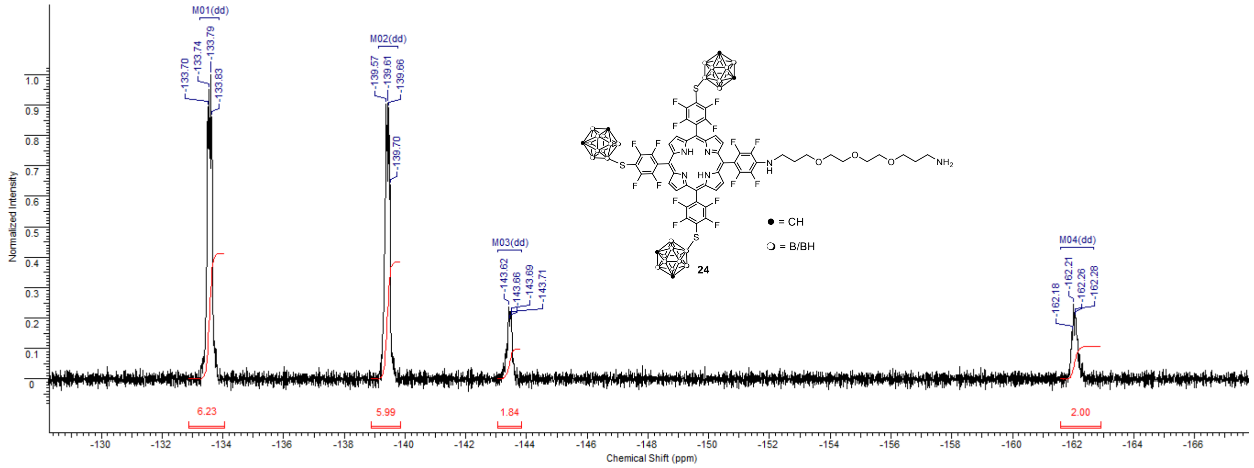

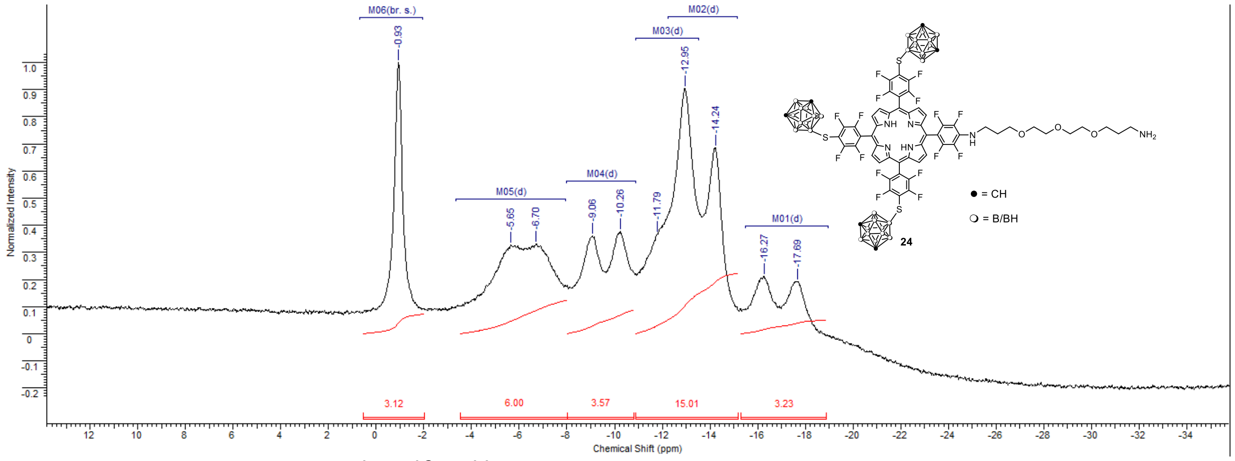

Figure S13. The ^{1}H, ^{19}F, ^{11}B NMR spectra of porphyrin **24** in $(CD_3)_2CO$.

16. The ^{1}H, ^{19}F and ^{11}B spectra of 5,10,15-tris{[4,4,4-(*m*-carboran-9'-yl)thio]-2,3,5,6-tetrafluorophenyl}-20-[4-((2'-sulfoethyl)amino)-2,3,5,6-tetrafluorophenyl]porphyrin (26)

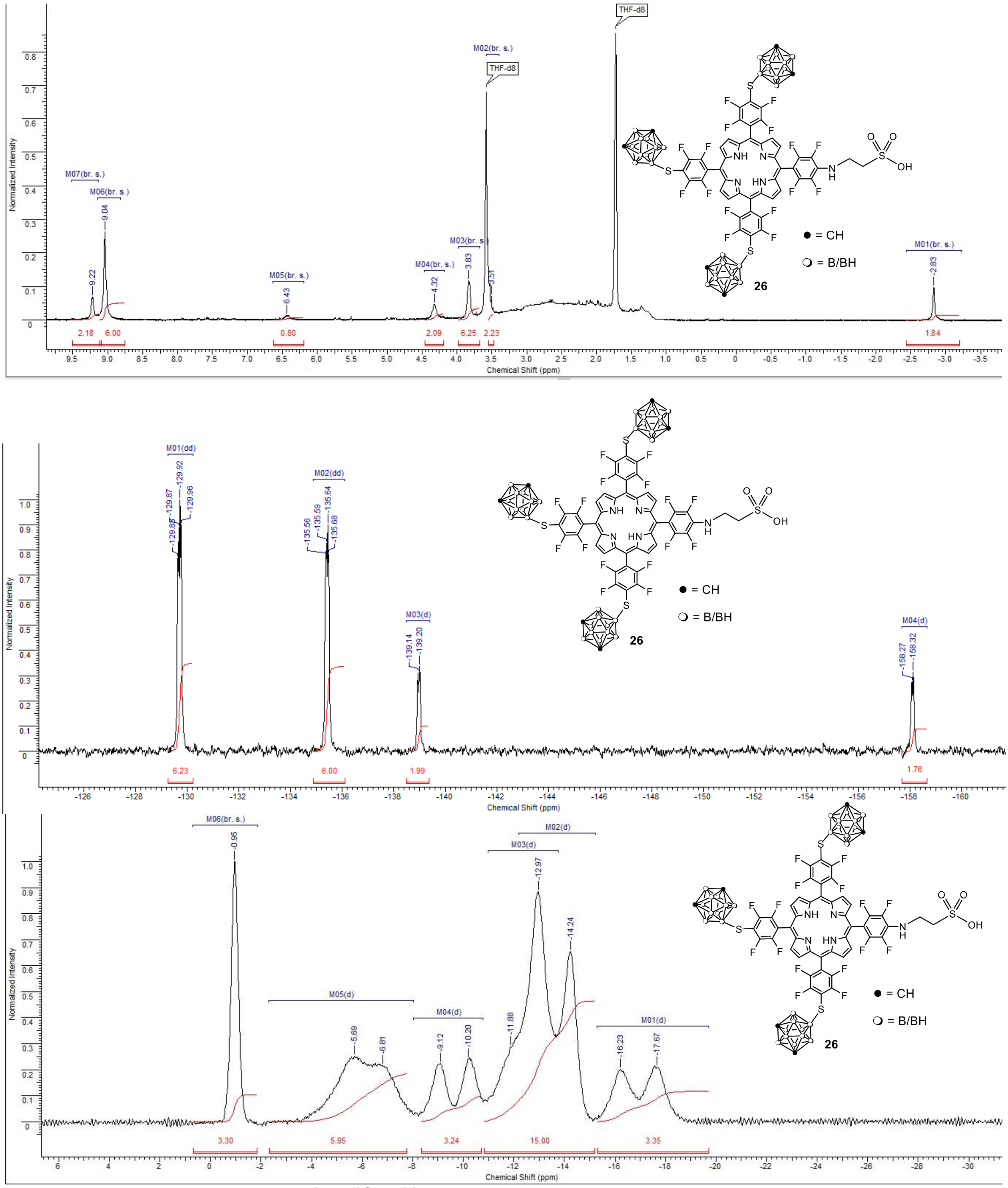

Figure S14. The ^{1}H, ^{19}F, ^{11}B NMR spectra of porphyrin **26** in THF-d_8.

17. MALDI-TOF mass spectrum for 5-(4-azido-2,3,5,6-tetrafluorophenyl)-10,15,20-tris(pentafluorophenyl)porphyrin (2)

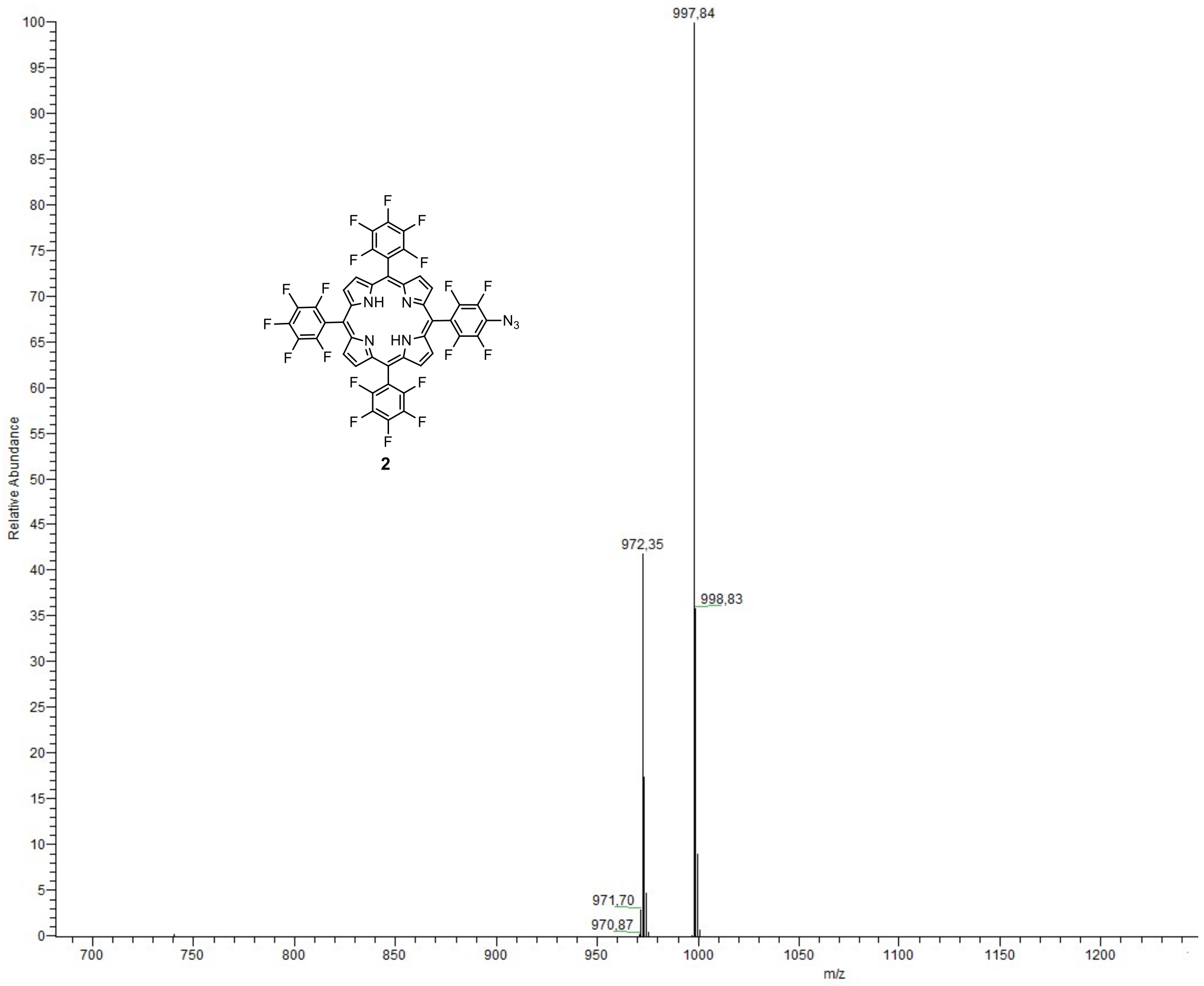

18. MALDI-TOF mass spectrum for 5-(4-amino-2,3,5,6-tetrafluorophenyl)-10,15,20-tris(pentafluorophenyl)porphyrin (3)

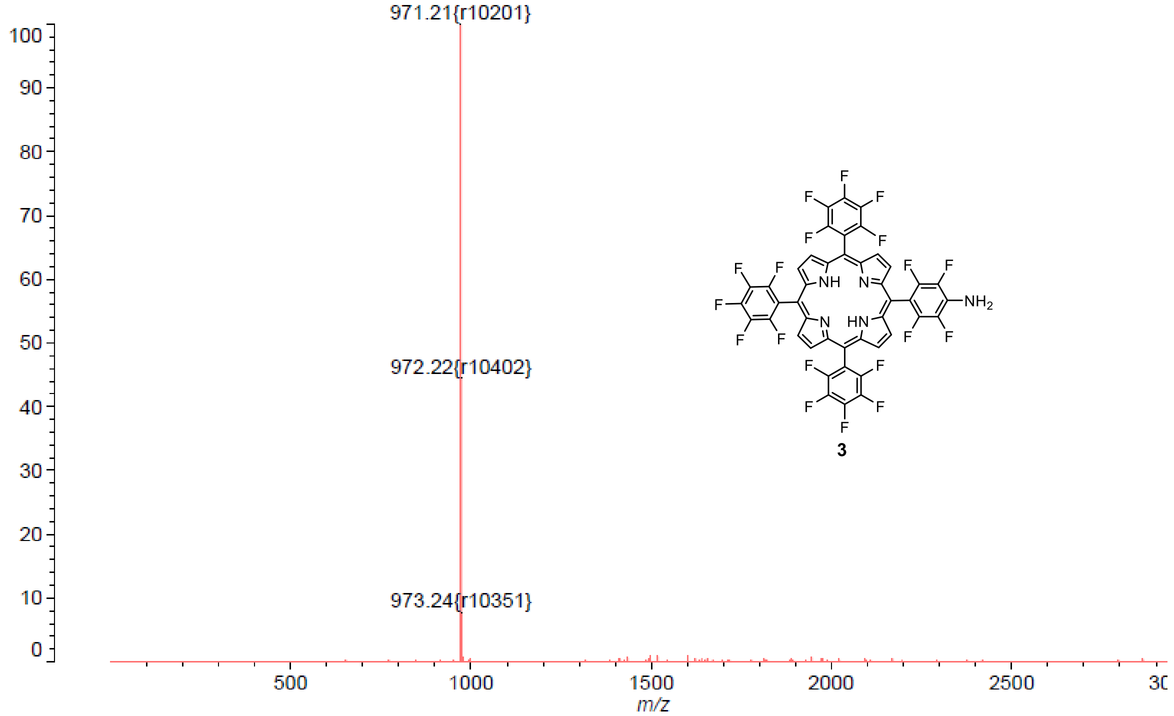

19. MALDI-TOF mass spectrum for 5-[4-amino-2,3,5,6-tetrafluorophenyl]-10,15,20-tris{[4-(*m*-carboran-9'-yl)thio]-2,3,5,6-tetrafluorophenyl}porphyrin (5)

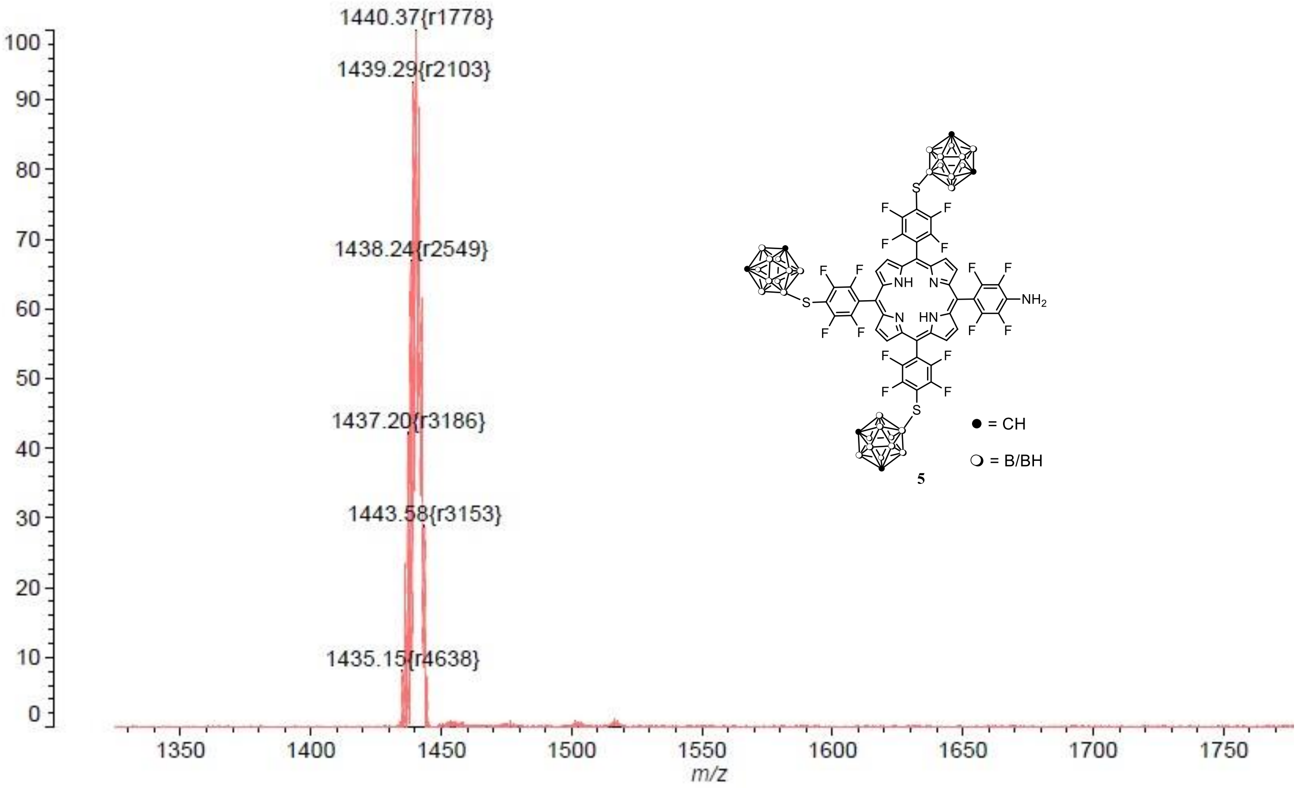

20. MALDI-TOF mass spectrum for 5, 10, 15-tris{[4-(*m*-carboran-9'-yl)thio]-2,3,5,6-tetrafluorophenyl}-20-(2,3,4,5,6-pentafluorophenyl]porphyrin (6)

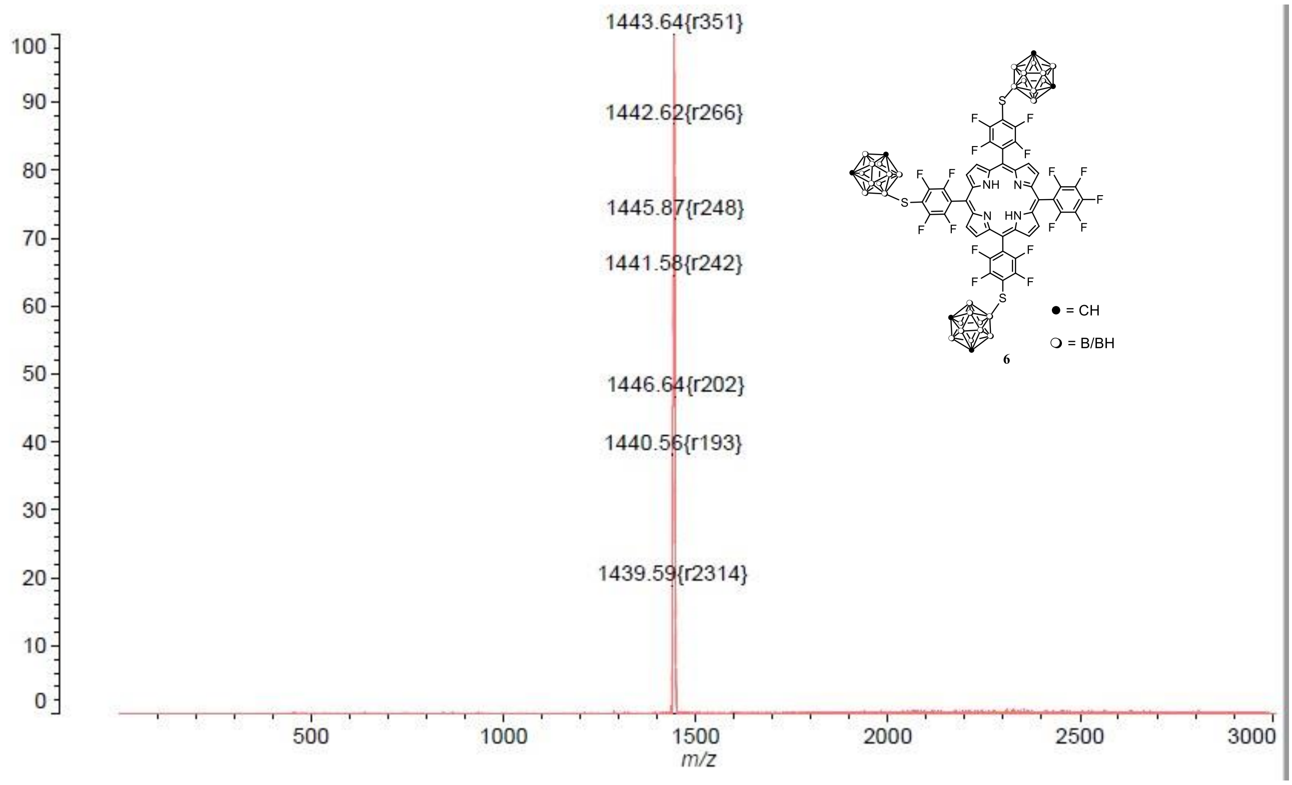

21. MALDI-TOF mass spectrum for 5-[4-azido-2,3,5,6-tetrafluorophenyl]-10,15,20-tris{[4-(*m*-carboran-9'-yl)thio]-2,3,5,6-tetrafluorophenyl}porphyrin (7)

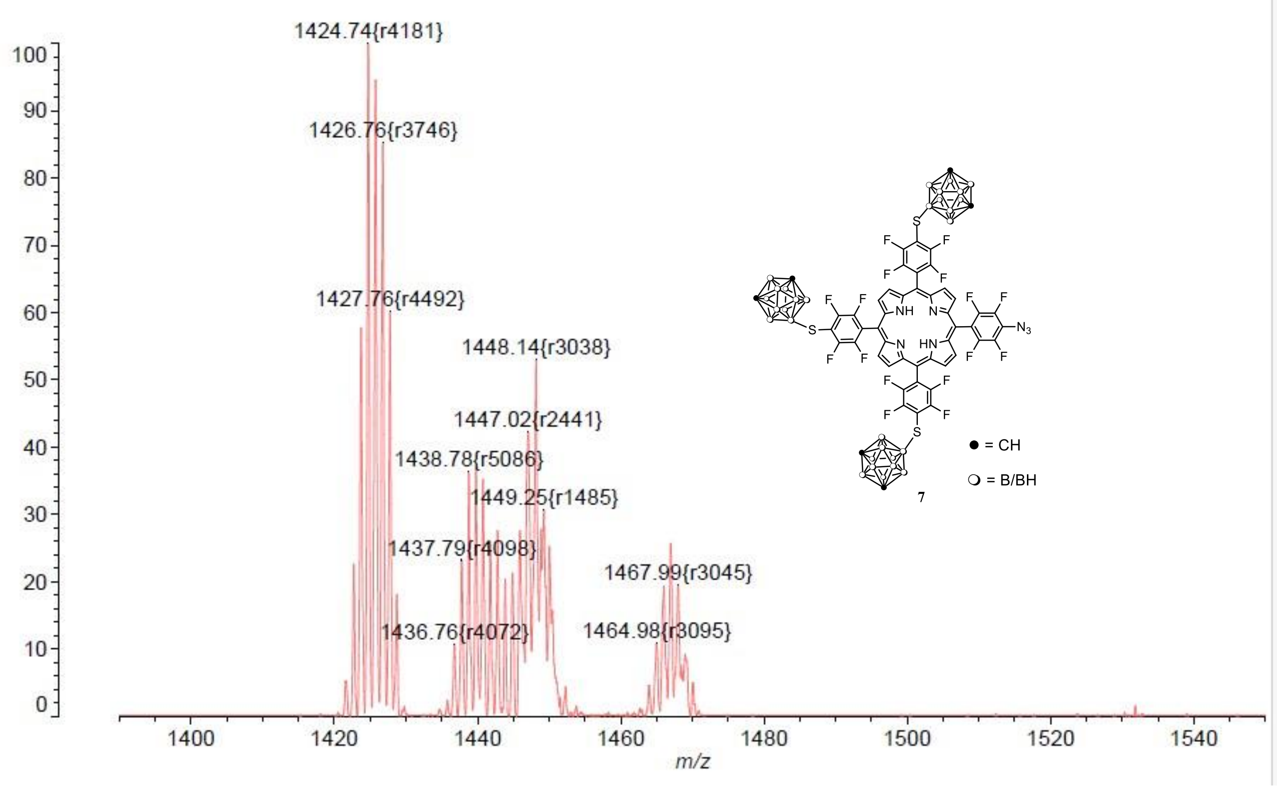

22. MALDI-TOF mass spectrum for 5,10,15-tris{[(*m*-carboran-9’-yl)thio]-2,3,5,6-tetrafluorophenyl}-20-{[4-(2’,5’-dioxo-2’,5’-dihydro-1*H*-pyrrol-1’-yl)benzamido]-2,3,5,6-tetrafluorophenyl}porphyrin (11)

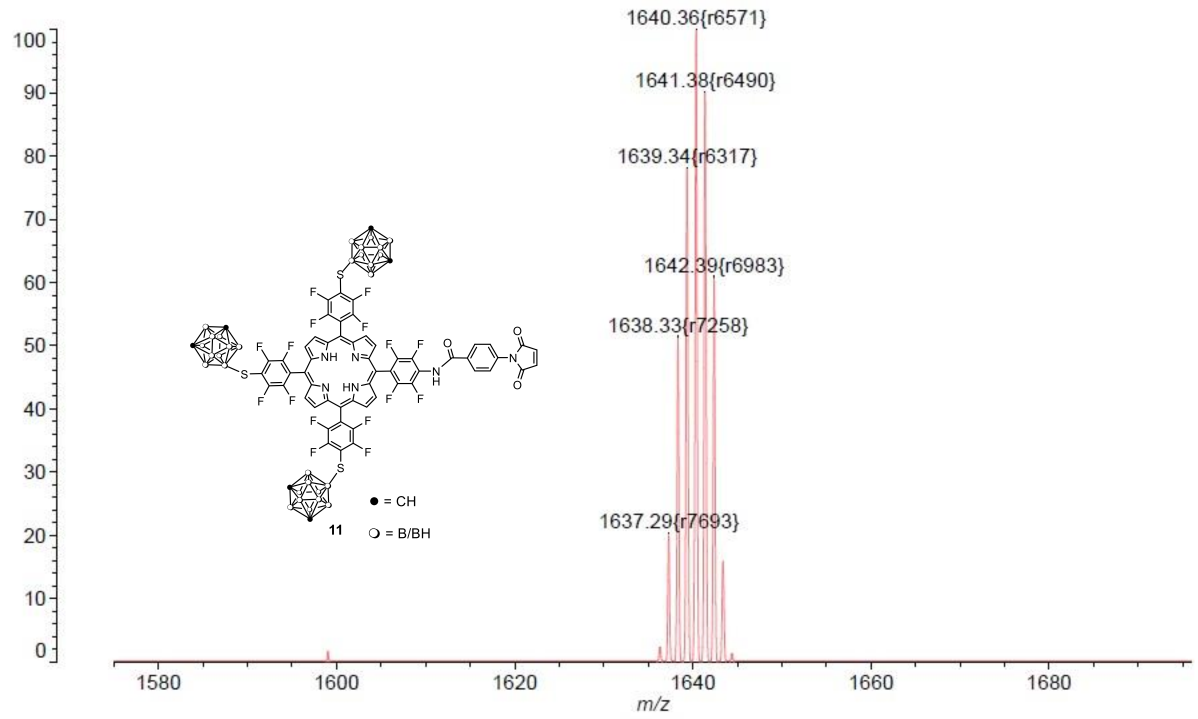

23. MALDI-TOF mass spectrum for 5,10,15-tris{[4,4,4-(*m*-carboran-9’-yl)thio]-2,3,5,6-tetrafluorophenyl}-20-[4-(2’-chloroacetamido)-2,3,5,6-tetrafluorophenyl]porphyrin (12)

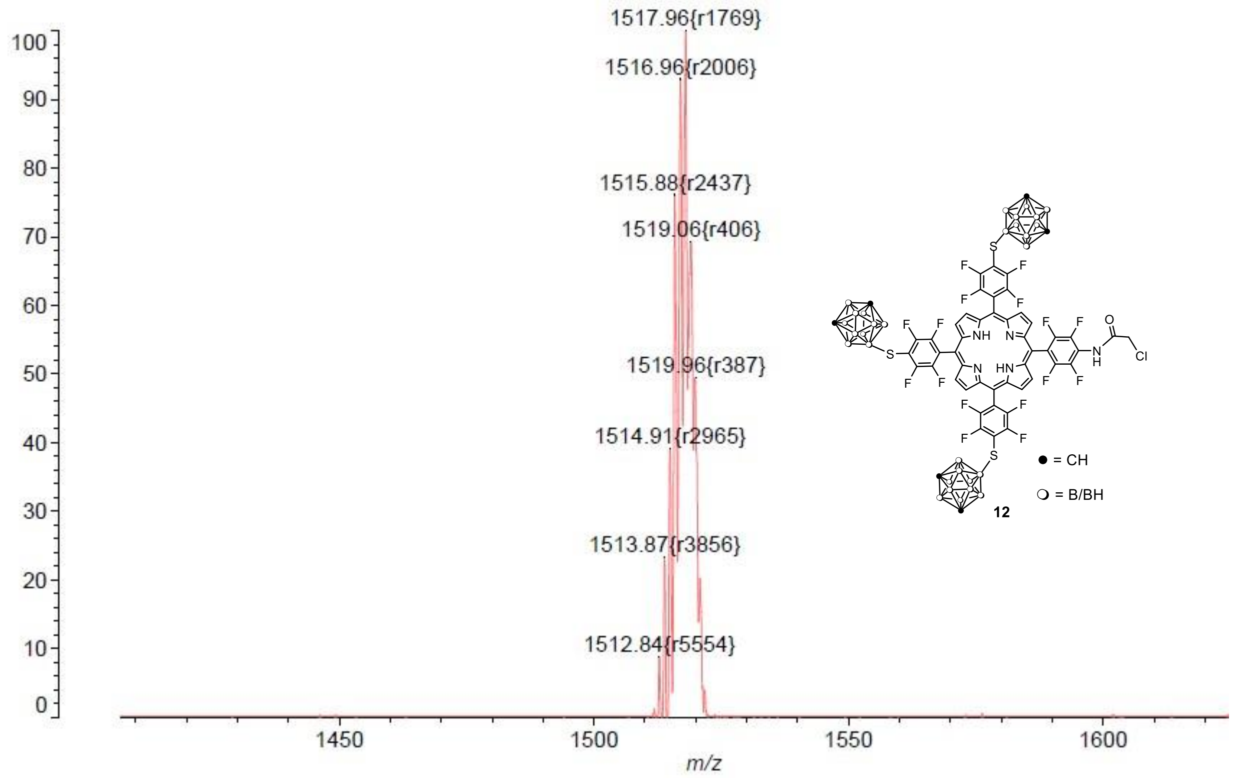

24. MALDI-TOF mass spectrum for 5,10,15-tris{[4,4,4-(*m*-carboran-9'-yl)thio]-2,3,5,6-tetrafluorophenyl}-20-[4-(2'-(4'-(4'-carboxybutyl)-2-oxohexahydro-*1H*-thieno[3,4-d]imidazol-1'-yl)acetamido)-2,3,5,6-tetrafluorophenyl]porphyrin (14)

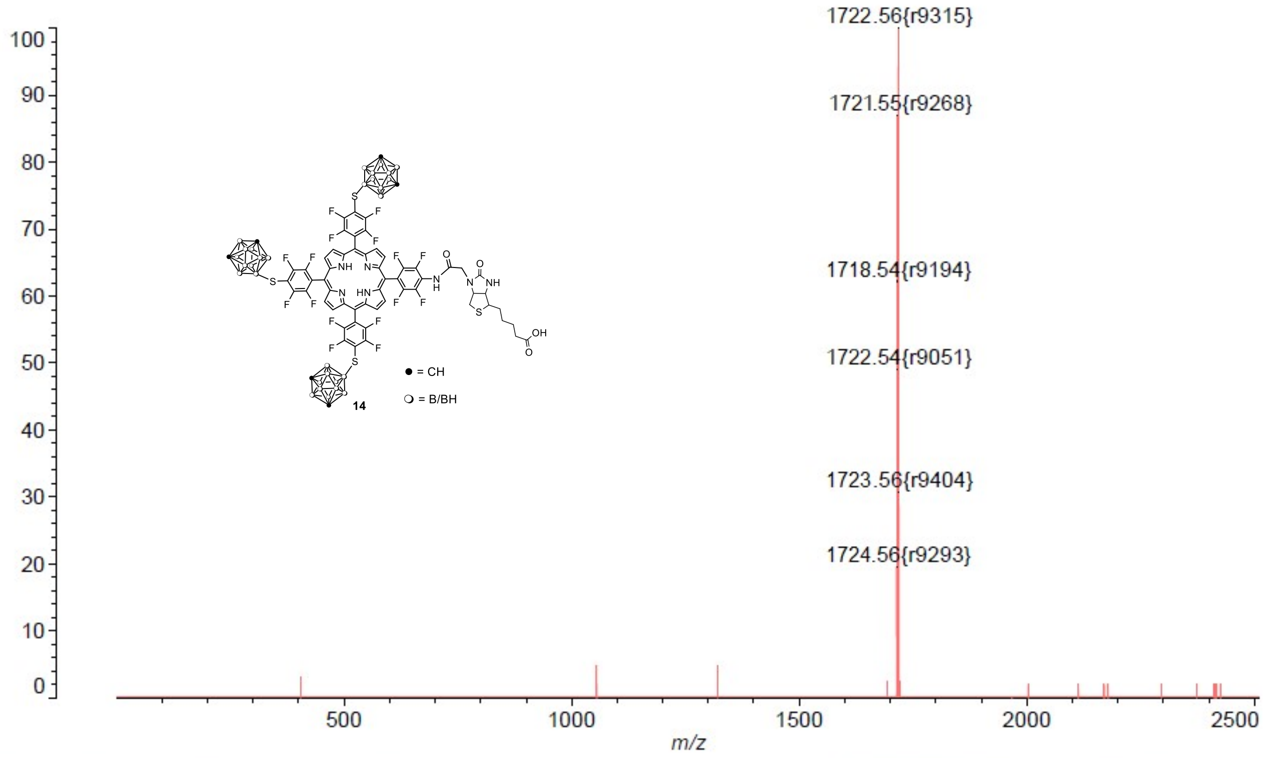

25. MALDI-TOF mass spectrum for 5,10,15-tris{[(*m*-carboran-9'-yl)thio]-2,3,5,6-tetrafluorophenyl}-20-{[(4-(2'-hydroxyethyl)thio]-2,3,5,6-tetrafluorophenyl}porphyrin (18)

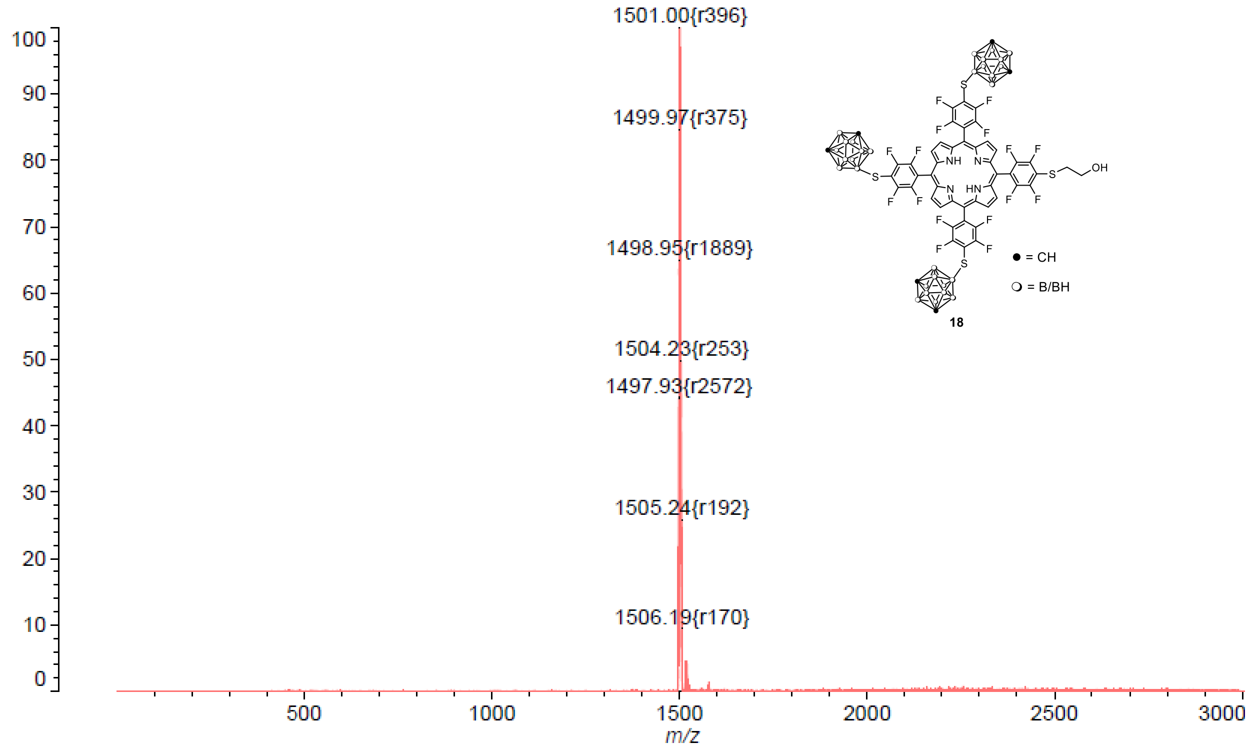

26. MALDI-TOF mass spectrum for 5-{[4-(2'-aminoethyl)thio]-2,3,5,6-tetrafluorophenyl}-10,15,20-tris{[4,4,4-(*m*-carboran-9'-yl)thio]-2,3,5,6-tetrafluorophenyl}porphyrin (19)

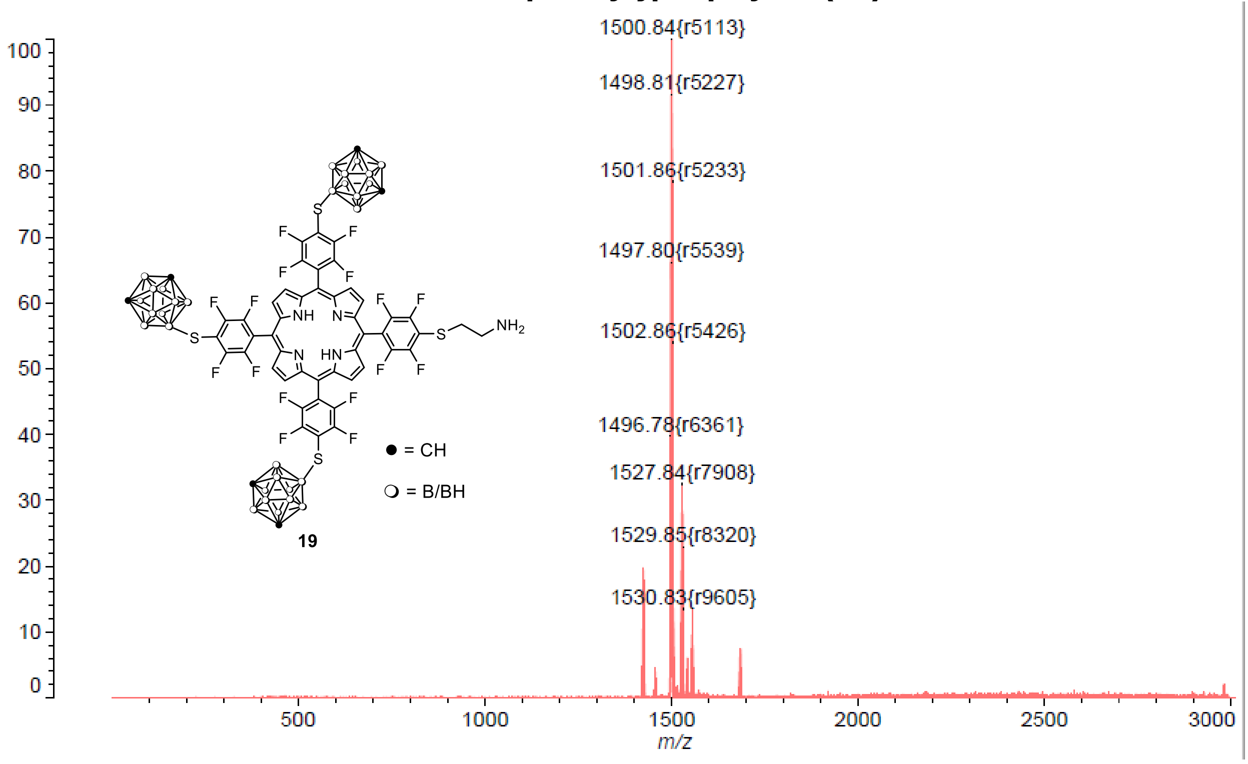

27. MALDI-TOF mass spectrum for 5,10,15-tris{[4,4,4-(*m*-carboran-9'-yl)thio]-2,3,5,6-tetrafluorophenyl}-20-{[(4-(3'-chloropropyl)thio]-2,3,5,6-tetrafluorophenyl]porphyrin (20)

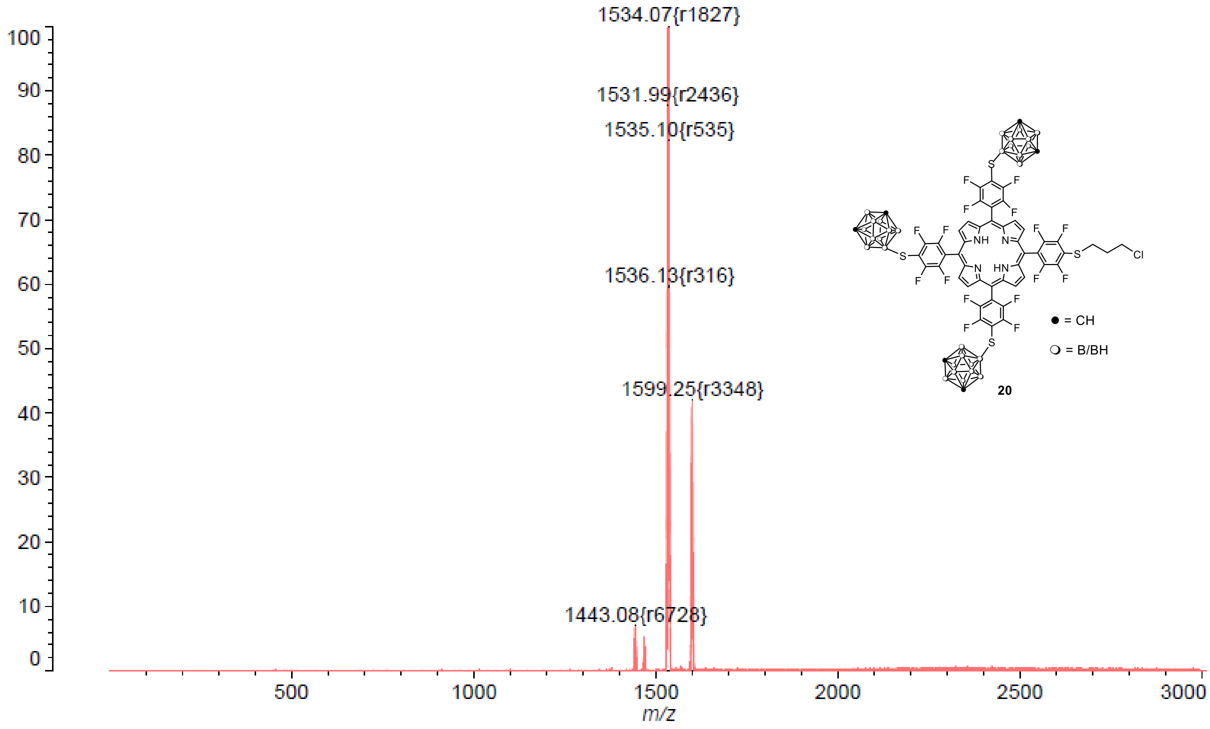

28. MALDI-TOF mass spectrum for 5-[(4-(2'-(2'-(2'-aminoethoxy)ethoxy)ethyl)amino)-2,3,5,6-tetrafluorophenyl]-10,15,20-tris{[4,4,4-(*m*-carboran-9'-yl)thio]-2,3,5,6-tetrafluorophenyl}porphyrin (23)

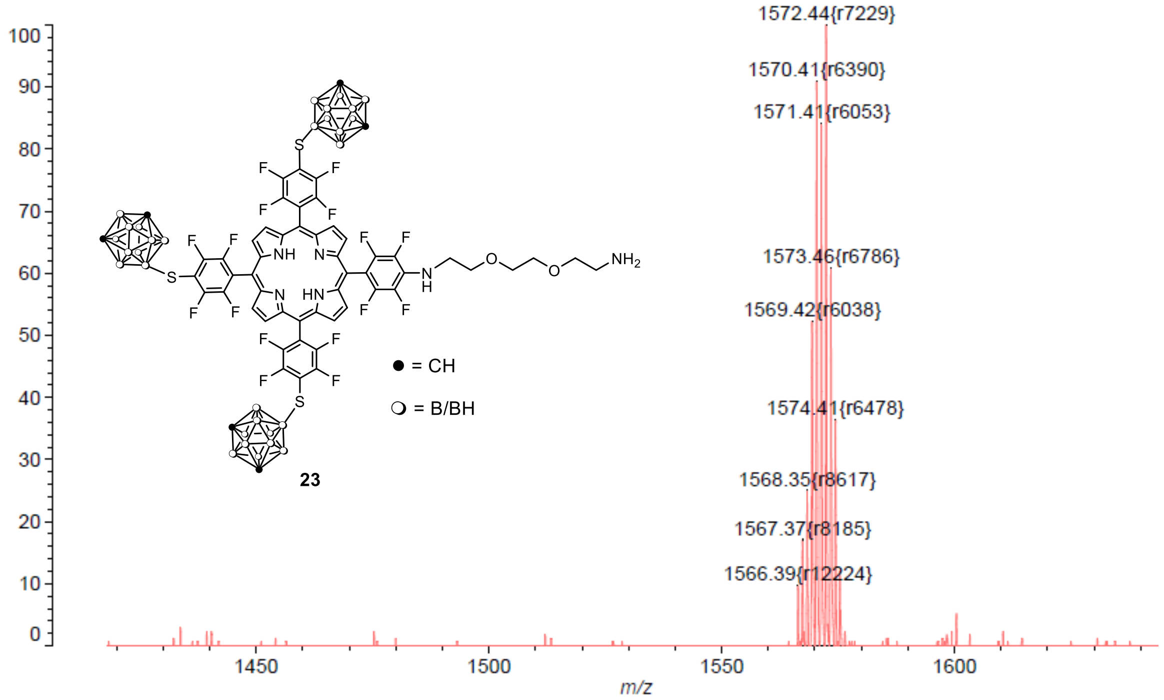

29. MALDI-TOF mass spectrum for 5-[(4-(((3'-(2'-(2'-(3'-aminopropoxy)ethoxy)ethoxy)propyl)amino)-2,3,5,6-tetrafluorophenyl]-10,15,20-tris{[4,4,4-(*m*-carboran-9'-yl)thio]-2,3,5,6-tetrafluorophenyl}porphyrin (24)

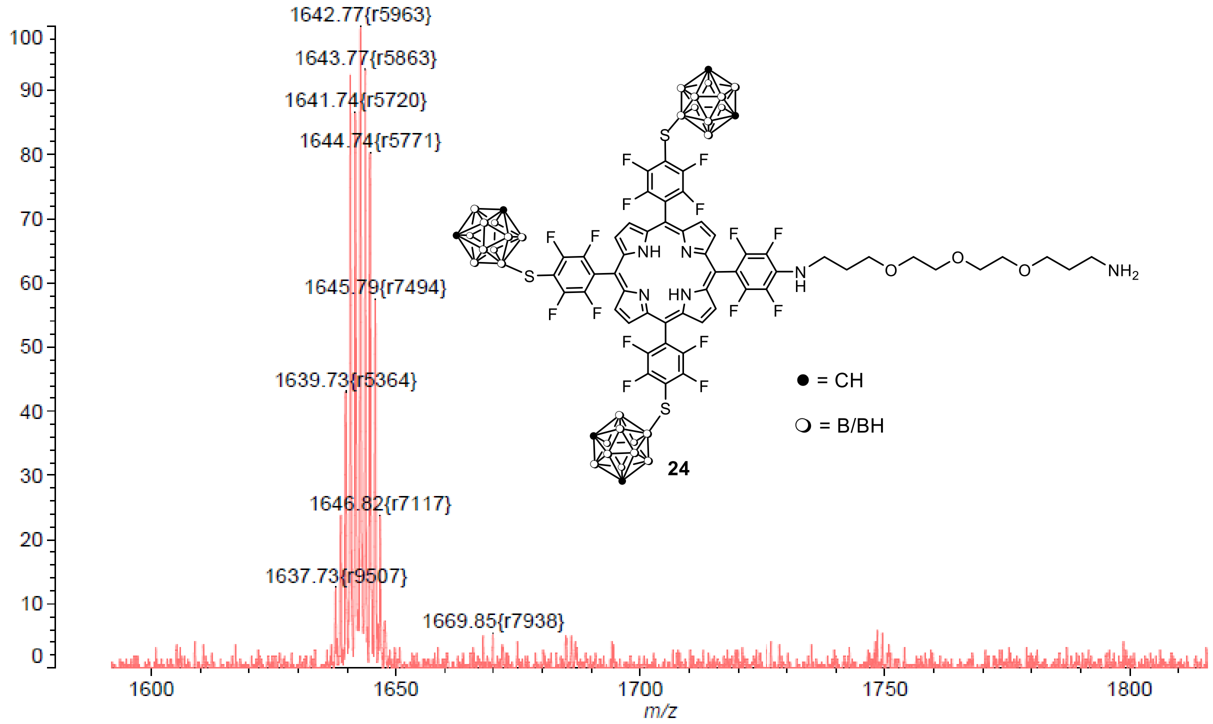

30. MALDI-TOF mass spectrum for 5,10,15-tris{[4,4,4-(*m*-carboran-9'-yl)thio]-2,3,5,6-tetrafluorophenyl}-20-[4-((2'-sulfoethyl)amino)-2,3,5,6-tetrafluorophenyl]porphyrin (26)

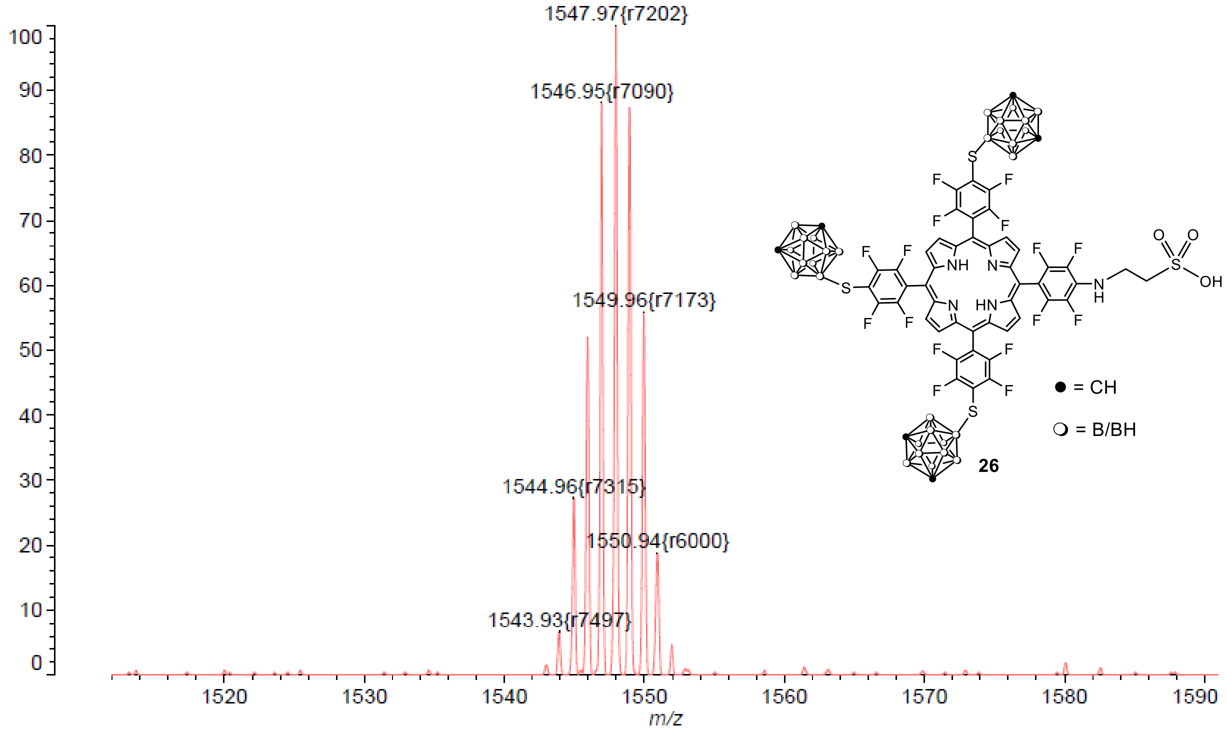

References

1. Lindsey, J.S.; Schreiman, I.C.; Hsu, H.C.; Kearney, P.C.; Marguerettaz A.M. *J. Org. Chem.* **1987**, *52,* 827–836.
2. Plešek, J.; Janoušek, Z.; Heřmánek, S. *Collect. Czech. Chem. Commun*. **1978**, *43*, 1332–1338.
